# Citric acid promoted melanin synthesis in B16F10 mouse melanoma cells, but inhibited it in human epidermal melanocytes and HMV-II melanoma cells via the GSK3β/β-catenin signaling pathway

**DOI:** 10.1371/journal.pone.0243565

**Published:** 2020-12-17

**Authors:** Siqi Zhou, Kazuichi Sakamoto

**Affiliations:** Faculty of Life and Environmental Sciences, University of Tsukuba, Tsukuba, Ibaraki, Japan; Wenzhou Medical University, CHINA

## Abstract

Melanin, a pigment synthesized by melanocytes in the skin, resists the damage caused by ultraviolet rays to cells. Citric acid, a well-known food additive, is commonly used as an antioxidant and is an important part of the tricarboxylic acid (TCA) cycle for energy production during cellular metabolism. Here, we aimed to investigate whether the addition of excess citric acid regulates melanin synthesis, and to delineate the underlying mechanism. First, we observed that citric acid exerts opposite redox effects on mouse and human cells. Interestingly, treatment with excess citric acid increased the melanin content in mouse cells but decreased it in human cells. Furthermore, the expression of factors important for melanin synthesis, such as microphthalmia-associated transcription factor (MITF), was also regulated by citric acid treatment—it was promoted in mouse cells and suppressed in human cells. Citric acid also impacted the upstream regulators of MITF, glycogen synthase kinase 3β (GSK3β), and β-catenin. Second, we determined the importance of GSK3β in the citric acid-mediated regulation of melanin synthesis, using a GSK3β inhibitor (BIO). To the best of our knowledge, this is the first study to show that citric acid regulates melanin synthesis via the GSK3β/β-catenin signaling pathway, and that equal amounts of exogenous citric acid exert opposing effects on mouse and human cells.

## Introduction

Melanin, an important pigment present in the skin of humans and animals, is a predominantly indolic polymer [1]. Melanin is produced by special pigment cells in the skin, called melanocytes, and is stored in membrane-bound organelles called melanosomes [2]. Commonly, the skin contains two types of melanin: red or yellow pheomelanin and black-brown eumelanin [2, 3]. Melanin is recognized as a photoprotective substance [3], with the ability to block ultraviolet radiation (UVR) [1, 4] and reduce the levels of UVR-induced cellular reactive oxygen species (ROS) and DNA photodamage [4, 5]. Abnormal melanin synthesis can cause various diseases, such as vitiligo and albinism (the lack of melanin synthesis) [1, 6]. Recent studies indicated that pheomelanin is one of the factors behind melanocyte carcinogenesis; pheomelanin synthesis and degradation processes induce the formation of cyclobutane pyrimidine dimers (CPD) in the nucleus, a kind of DNA damage, and increase the risk of carcinogenesis [7, 8]. Therefore, melanin synthesis should be studied for health and cosmetic purposes.

The process of melanin synthesis has been extensively studied, and the amino acid tyrosine is considered the starting material for melanin synthesis. Tyrosinase, which catalyzes the hydroxylation of tyrosine, is the most important rate-limiting enzyme regulating melanin synthesis [9–11]. Tyrosine is initially oxidized by tyrosinase to form dopaquinone (DQ), and DQ spontaneously forms dopachrome and L-3,4-dihydroxyphenylalanine (L-DOPA) in the melanosomes. Next, L-DOPA is oxidized by tyrosinase to again form DQ, while dopachrome decomposes to form dihydroxyindole (DHI) and dihydroxyindole-2-carboxylic acid (DHICA). Finally, DHI and DHICA are catalyzed to form melanin [12]. Tyrosinase expression is regulated by the transcription factor, microphthalmia-associated transcription factor (MITF, contains a basic helix- loop-helix-leucine zipper structure), which is, in turn, generally regulated by the melanocortin-1 receptor (MC1R)-activated cyclic adenosine monophosphate (cAMP) protein kinase A (PKA), and by the cAMP response element-binding protein (CREB) signaling pathway [9–11]. Recent studies suggested that MITF is also regulated by some other upstream signaling pathways. For instance β-Catenin, a key downstream effector of the Wnt signaling pathway was reported to regulate the expression of MITF [13]. In the absence of Wnt signaling, a multiprotein complex triggered by GSK3β is formed, phosphorylating β-catenin and inducing its degradation via the proteasome. However, in the presence of Wnt signaling, the GSK3β-dependent phosphorylation of β-catenin is blocked [13, 14]. As a part of post-transcriptional regulation, the MEK/ERK signaling cascade induces MITF phosphorylation at S79, which subsequently causes the degradation of MITF via the ubiquitin-proteasome pathway [15]. In addition to the Wnt/β-catenin signaling pathway, GSK3β can directly modulate MITF activity. Previous studies have shown that GSK3β can phosphorylate MITF at the S298 site, facilitating the binding of MITF to the tyrosinase promoter [16, 17].

Citric acid (2-hydroxy-1,2,3-propane-tricarboxylic acid) is a weak organic acid widely found in citrus fruits, such as lemon, grapefruit, tangerine, and orange. It is usually used as a food, drink, and cosmetic additive [18, 19]. Citric acid is an intermediate of the tricarboxylic acid (TCA) cycle, which plays a central role in energy metabolism, macromolecule synthesis, and the maintenance of the cellular redox balance. The TCA cycle consists of a series of biochemical reactions in the mitochondrial matrix, enabling aerobic biological oxidation of fuel resources and providing energy, macromolecules, and the redox balance. The perturbation of the TCA cycle results in various diseases [20]. Of note, the TCA cycle generates many other organic acids, such as pyruvic acid, succinic acid, and malic acid. Previously, we have shown that the addition of exogenous pyruvate can inhibit the synthesis of melanin in B16F10 mouse melanoma cells [21]; hence, we speculated that the other organic acids may exert the same effect. After analyzing and screening each organic acid, we observed that contrary to the inhibitory effect of pyruvic acid, citric acid promoted melanin synthesis in B16F10 mouse melanoma cells. This result is consistent with the results of previous studies [22]; of note, these studies did not focus on the mechanism via which citric acid regulates melanin synthesis. Many previous studies have shown that citric acid can reduce the damage caused by oxidative stress, and possesses anti-inflammatory properties [18]. Recent studies also showed that citric acid can be used as an anti-tumor agent [23, 24]. Therefore, the mechanism via which citric acid regulates the synthesis of melanin, and the effect of citric acid on human cell lines should be investigated.

In this study, we used B16F10 mouse melanoma cells, HMV-II human melanoma cells, and normal human epidermal melanocytes (HEM) to simultaneously determine the effect of citric acid on melanogenesis, and to investigate the upstream signaling cascade(s) regulating the activity of MITF after citric acid treatment. This is the first study to show that equal amounts of citric acid regulate melanin synthesis differently in human and mouse cells.

## Materials and methods

### Materials

B16F10 and HMV-II melanoma cell lines were obtained from the RIKEN Institute, Physical and Chemical Research Cell Bank (Tsukuba, Japan). Citric acid powder, Roswell Park Memorial Institute (RPMI)-1640 medium, and 3-(4,5-dimethylthiazol-2-yl)-2,5-diphenyltetrazolium bromide (MTT) were purchased from Sigma-Aldrich (St. Louis, MO, USA). Anti-tyrosinase antibodies were purchased from Santa Cruz Biotechnology (Santa Cruz, CA, USA). Antibodies against MITF, GSK3β, phospho-GSK3β, β-catenin, and total β-actin were obtained from Cell Signaling Technology (Tokyo, Japan). The normal human epidermal melanocyte (NHEM) cell line and the special medium kit (DermaLife^R^ Basal Medium) were obtained from KURABO (Osaka, Japan).

### Cell culture

B16F10 and HMV-II cells were cultured in RPMI-1640 medium supplemented with 10% fetal bovine serum; NHEM cells were cultured in the special culture medium at 37°C in the presence of 5% CO_2_. After a primary culture from frozen stocks in 10-cm dishes, the cells were seeded at densities of 1.0 × 10^5^ cells/ well in 6-well-plates, 3.0 × 10^3^ cells/well in 24-well-plates, or 1.0 × 10^3^ cells/well in 96-well-plates. The cells were allowed to attach to the bottom of the plates for 24 h before subsequent experiments.

### MTT assay

Cells were cultured in 24-well-plates. After a 48-h treatment with citric acid, the medium was replaced with fresh medium containing 500 μg/ml MTT reagent, and the cells were incubated at 37°C for another 4 h. Isopropanol with 0.04 M HCl solution was added and the plates were maintained at 25°C for 5 min. The colored solution was transferred to a 96-well assay plate, and the absorbance (isopropanol with 0.04 M HCl solution was used as the blank) at 570 nm was measured using a microplate reader (Tecan, Kawasaki, Japan).

### Cell growth curve

The cells were seeded into six 24-well-plates. After 24 h of cell attachment, the medium in each well was exchanged with fresh media containing 2 mM, 4 mM, and 6 mM citric acid. Medium with citric acid was replaced every 2 days, and the cell numbers were counted each day for B16F10, and after every 2 days for human cell lines.

### Detection of intracellular ROS

Intracellular ROS was detected using the cellular reactive oxygen species detection kit as per the manufacturer’s instructions (Abcam, Tokyo, Japan). The cells were treated with or without citric acid for 2 h, washed twice with washing buffer, and treated with 2',7'-dichlorodihydrofluorescein diacetate (DCFDA) for 1 h. The cells were washed again with washing buffer to remove DCFDA, and fluorescence (excitation/emission = 495/529 nm) was measured using a microplate reader.

### 2,2-Diphenyl-1-picrylhydrazyl (DPPH) assay

DPPH, purchased from Sigma-Aldrich (St. Louis, MO, USA), was dissolved in ethanol to obtain a final concentration of 0.04 mg/ml. Ascorbic acid (vitamin C) and BHT were dissolved in 70% ethanol to the concentrations of 1 mg/ml and 2 mg/ml, respectively (only for this assay, citric acid was prepared in 70% ethanol). Ascorbic acid was diluted to 10, 20, 50, 100, and 200 μg/ml (group A), while BHT (group B) and citric acid (group C) were diluted to 100, 200, 500, 1000, and 2000 μg/ml. Twenty microliter samples from groups A, B, and C were individually added into another 96-well assay plate, followed by the addition of 180 μl DPPH solution in each well. The plate was incubated at room temperature for 30 min in the dark, and the absorbance was measured at 517 nm using a microplate reader. Ethanol (70%) was used as the blank, and 20 μl 70% ethanol plus 180 μl DPPH was used as the positive control. The scavenging activity was calculated as follows:

Scavengingactivity=(1–SampleOD/PositivecontrolOD)×100%

### Measurement of the melanin content

The melanin content was determined as described previously. Cells were seeded in a 6-well plate. After citric acid treatment from days 1 to 6, the cells were collected via trypsinization. After centrifugation at 1000 × *g* for 5 min, the supernatant was removed, and the cell pellet was resuspended in 100 μl of 1 N NaOH. After heating at 80°C for 1 h, the absorbance at 405 nm was measured on a microplate reader. The melanin content was normalized to that of total protein (determined using the Pierce™ bicinchoninic acid (BCA) protein assay kit, as per the manufacturer’s instructions; Thermo Fisher Scientific, Waltham, MA, USA).

### Tyrosinase activity

Tyrosinase activity was estimated by measuring the rate of L-DOPA oxidation. B16F10, HMV-II, and HEM cells were incubated in 6-well-plates and treated for 4 or 6 days. The cells were collected via trypsinization, suspended in phosphate buffer containing 10% Triton-X, sonicated, and centrifuged at 14,000 rpm for 20 min. The supernatant was assayed for total protein, and 50 μg protein was mixed with 2 μl 10% (m/v) L-DOPA (Sigma Aldrich, Sr. Louis, MO, USA) in phosphate buffer at 37°C. Subsequently, absorbance was measured at 475 nm. Tyrosinase activity was calculated using the following formula:

$$Tyrosinase\;activity=sample\;OD_{475}/control\;OD_{475}$$

### Western blotting

Cells (2.5 × 10^5^) were seeded in a 6-cm dish. After treatment with citric acid for different durations, the cells were suspended in radioimmunoprecipitation assay buffer, sonicated, and centrifuged at 10,000 × *g* for 10 min. The supernatants were collected (protein quantification was performed using the Pierce^TM^ BCA protein assay kit, as per the manufacturer’s instructions), and 20 μg protein from each sample was separated using sodium dodecyl sulfate-polyacrylamide gel electrophoresis (SDS-PAGE). The proteins were transferred onto a polyvinylidene difluoride membrane, which was blocked for 1 h in 2% bovine serum albumin (BSA) and then incubated overnight at 4°C with primary antibodies against MITF, tyrosinase, GSK3β, phospho-GSK3β, and β-catenin. This was followed by incubation for 60 min at room temperature with the appropriate secondary antibodies. The membrane was stained with LuminoGLO (Cell Signaling Technology), and protein bands were visualized using an AE-9300H EZ-Capture MG imager (ATTO Corporation, Tokyo, Japan).

### Statistical analysis

The results are reported as mean ± standard deviation. Group means were compared using the Student’s t-test, and differences were considered significant at P < 0.05.

## Results

### Citric acid inhibited melanocytes and melanoma cell proliferation

**Fig 1:**
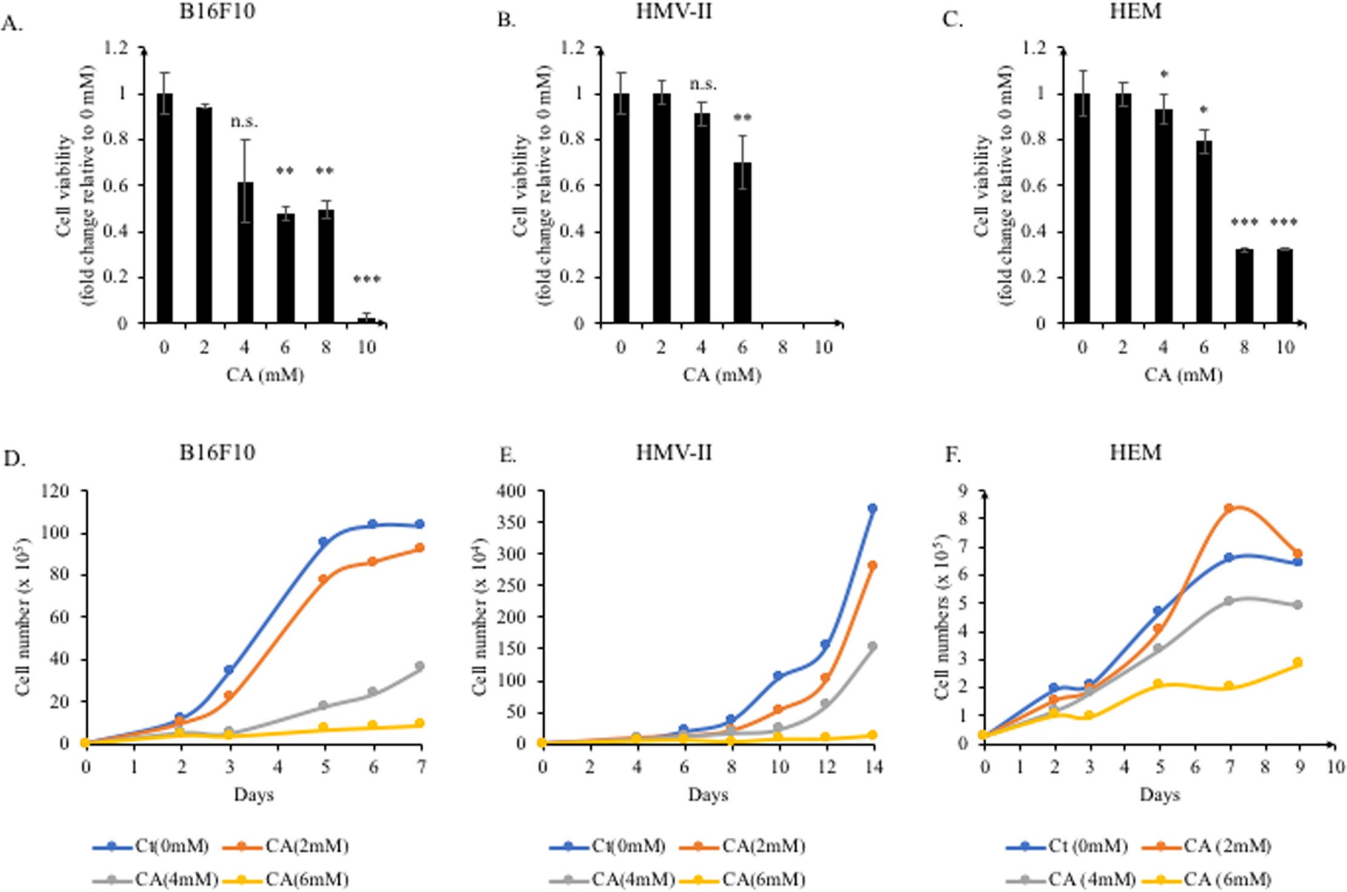
The effect of citric acid on the viability and proliferation of B16F10, HMV-II, and HEM cells. B16F10, HMV-II, and HEM cells were treated with citric acid (CA). (A−C) After treatment with CA (2, 4, 6, 8, and 10 mM) for 2 days, the MTT assay was performed to assess the cell viability. CA dose-dependently decreased the cell viability. (D−F) Low-density cell cultures (3000 cells/well) were established for prolonged treatment. Cell numbers were determined daily, for B16F10 cells, and every two days for HMV-II and HEM cells. The cell growth curves were plotted directly using the cell numbers. CA decreased cell proliferation in all cell lines. Results are presented as mean ± SD. Statistical analysis was performed using the Student’s t-test versus the non-treated groups (0 mM), n ≥ 3, *P < 0.05; **P < 0.01; ***P < 0.001, n.s. not significant.

First, we determined the concentration of citric acid that supported cell viability. Concentrations ranging from 2 mM to 10 mM were used to treat mouse melanoma cells (B16F10), human melanoma cells (HMV-II), and normal human epidermal melanocytes (HEM). After 2 days of treatment, cell viability was assessed using the MTT assay. Citric acid concentrations >6 mM were toxic for all three cell lines (Fig 1A–1C); no live cells were detected in HMV-II cells treated with 8 mM and 10 mM citric acid (Fig 1B). The viability of mouse melanoma cells (B16F10) treated with <6 mM citric acid was considerably reduced (Fig 1A), while the effect on human cells was not clear.

Next, we also tested the effect of citric acid on cell proliferation. In total, 3,000 cells of each cell line were used to start the culture; the cells were maintained in culture until the non-treatment groups reached confluency. In all treatment-conditions, floating cells did not appear in the culture medium. We observed that citric acid dose-dependently inhibited cell proliferation (Fig 1D–1F), with a stronger inhibitory effect on mouse cells. Owing to its strong cell proliferation inhibitory effect, we used <4 mM citric acid in the subsequent experiments.

### Citric acid inhibited ROS generation in human cells but promoted it in mouse cells

**Fig 2:**
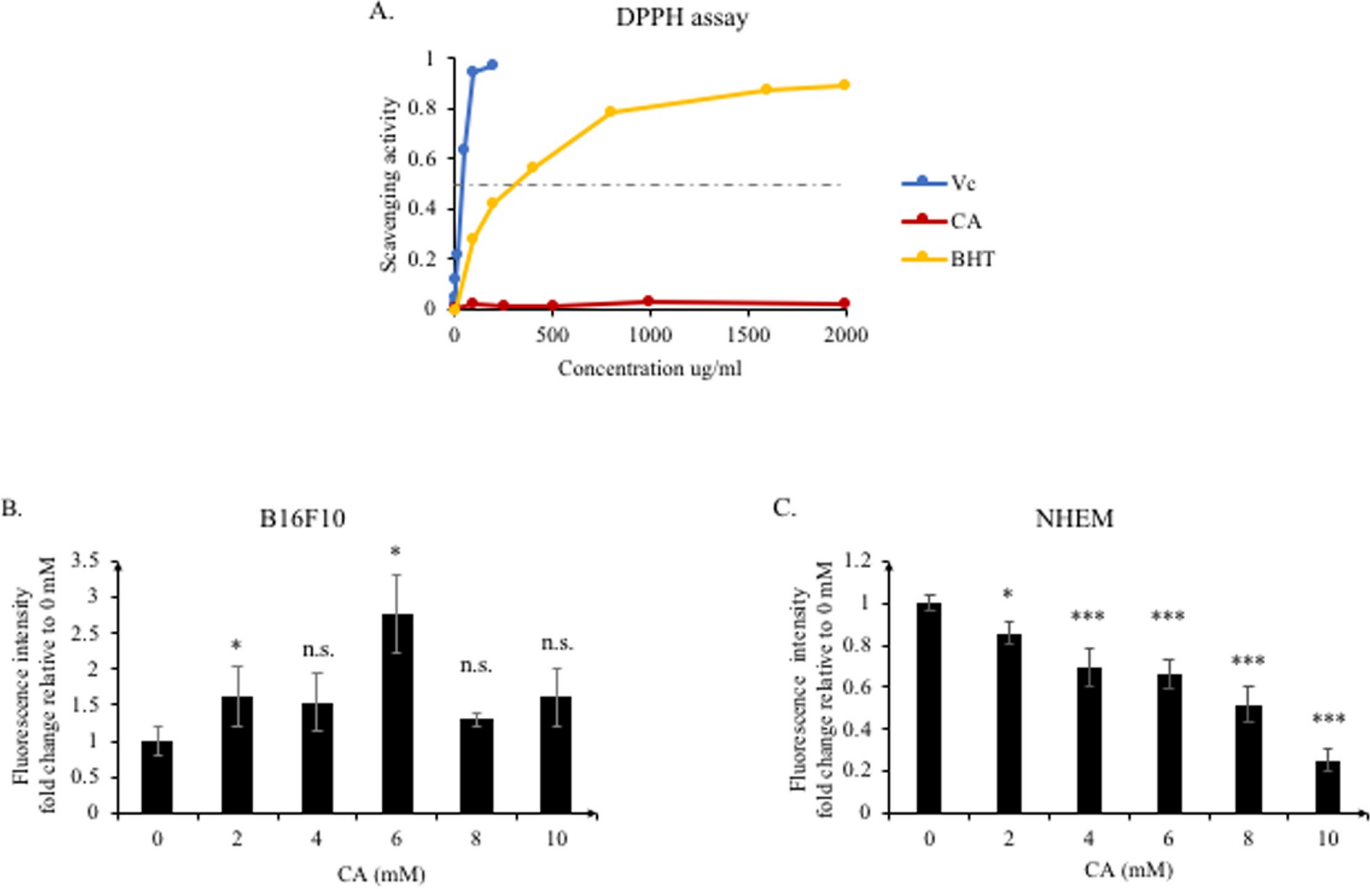
Extracellular and intracellular scavenging activity of citric acid. (A) The DPPH assay was performed with ascorbic acid (Vc), BHT, and citric acid (CA). CA did not affect the scavenging activity outside the cell. (B−C) B16F10 and NHEM cells were treated with CA for 2 h. CA increased the ROS levels in B16F10, but dose-dependently decreased ROS in NHEM cells. Results are presented as mean ± SD. Statistical analysis was performed using the Student’s t-test versus the non-treated groups (0 mM), n ≥ 3, *P < 0.05; ***P < 0.001, n.s. not significant.

As mentioned previously, citric acid possesses antioxidant activity. Therefore, we used the DPPH and DCFDA assays to confirm the intracellular and extracellular antioxidant capacities of citric acid, respectively. First, we used ascorbic acid (Vitamin C, Vc) and butylated hydroxytoluene (BHT) as the positive antioxidant controls to test the extracellular chemical antioxidant capacity of citric acid. As shown in Fig 2A, compared to ascorbic acid and BHT, citric acid showed almost no chemical antioxidant capacity.

We next treated mouse and human cells with citric acid, and detected the content of intracellular ROS generated, using the DCFDA reagent after 2 h. Surprisingly, the opposite results were obtained with mouse and human cells. Citric acid gradually reduced the amount of ROS in human cells in a dose-dependent manner (Fig 2C). On the other hand, in mouse cells, citric acid promoted the accumulation of intracellular ROS, irrespective of the dose used (Fig 2B).

### Citric acid promoted melanogenesis in mouse cells but inhibited it in human cells, via the regulation of tyrosinase activity

**Fig 3:**
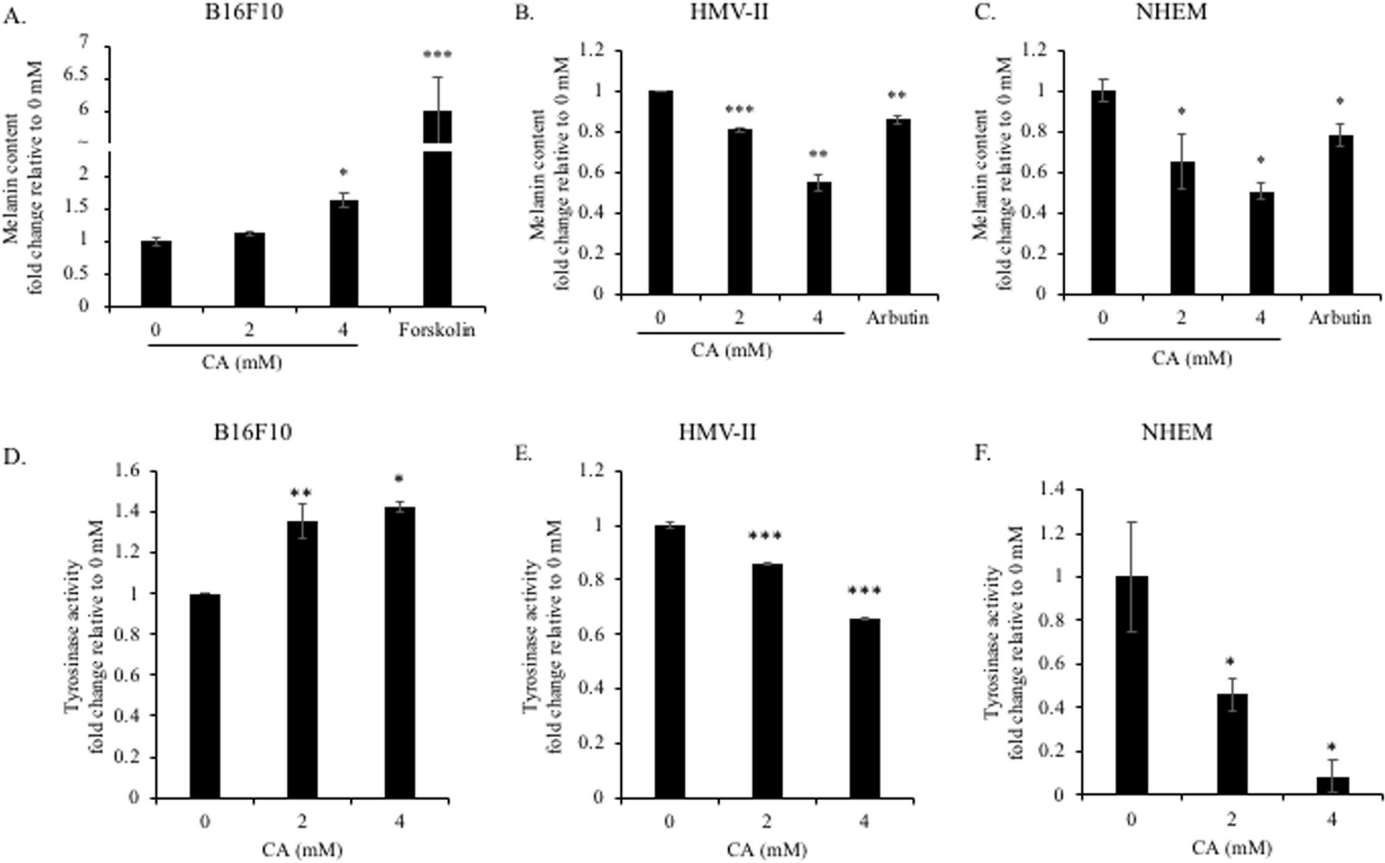
Effect of citric acid on melanogenesis and tyrosinase activity in B16F10, HMV-II, and HEM. Cells were treated with citric acid (CA) (4 days for B16F10 and 6 days for HEM and HMV-II). (A−C) The intracellular melanin content was assessed; the amount was normalized to that of the total protein. Forskolin and arbutin were used as the positive and negative controls of melanogenesis, respectively. The melanin content increased in mouse cells (B16F10) and decreased in human cells (HMV-II and HEM). (D−F) After treatment under the same conditions used for the determination of melanin synthesis, cells were collected and lysed, and tyrosinase activity was measured using L-DOPA as the substrate. Consistent with the results of melanin content analysis, tyrosinase activity increased in mouse cells and decreased in human cells. Results are presented as mean ± SD. Statistical analysis was performed using the Student’s t-test versus the non-treated groups (0 mM), n ≥ 3, *P < 0.05; **P < 0.01; ***P < 0.001.

To detect the effect of citric acid on melanogenesis, we determined the intracellular melanin content after citric acid treatment. Cells were cultured to confluency to avoid changes in melanin synthesis due to the differences in the proliferation rates of human and mouse cells (B16F10, 4 days; HMV-II and HEM, 6 days). Forskolin [25] and arbutin [26] were used as the positive controls for up- and down-regulating melanogenesis, respectively. In B16F10 mouse melanoma cells, forskolin considerably promoted the synthesis of melanin (Fig 3A). Although citric acid did not promote melanogenesis to the same extent as forskolin, it promoted melanin synthesis in a dose-dependent manner (Fig 3A). In contrast, arbutin reduced melanin synthesis in both HEM and HMV-II human cells (Fig 3B and 3C). Citric acid also reduced melanin synthesis in human cells in a dose-dependent manner (Fig 3B and 3C).

As tyrosinase is an important rate-limiting enzyme for melanin synthesis, its catalytic activity can directly reflect the efficiency of melanin synthesis. Hence, we assessed the intracellular activity of tyrosinase. After citric acid treatment for the same duration as in the melanin synthesis experiments, the cells were collected and disrupted, and the supernatants were collected. L-DOPA was used as the catalytic substrate of tyrosinase. The amount of melanin synthesized directly reflects the catalytic activity of tyrosinase. Consistent with the results of the melanin synthesis experiments, citric acid promoted the catalytic activity of tyrosinase in B16F10 mouse melanoma cells (Fig 3D) and inhibited the activity of tyrosinase in the two human cell lines (Fig 3E and 3F). Therefore, we concluded that citric acid impacts the intracellular synthesis of melanin via the regulation of tyrosinase activity.

### Citric acid oppositely regulated the expression of tyrosinase via the GSK3β and β-catenin signaling pathways in human and mouse cells

As citric acid regulated the activity of tyrosinase, we further studied the upstream regulatory pathways. It is well known that MITF is an important transcription factor regulating melanin synthesis, directly controlling the expression of the tyrosinase-encoding gene. Furthermore, MITF expression and activity are also regulated by other upstream factors. Here, we focused on the regulation of MITF by GSK3β and β-catenin.

**Fig 4:**
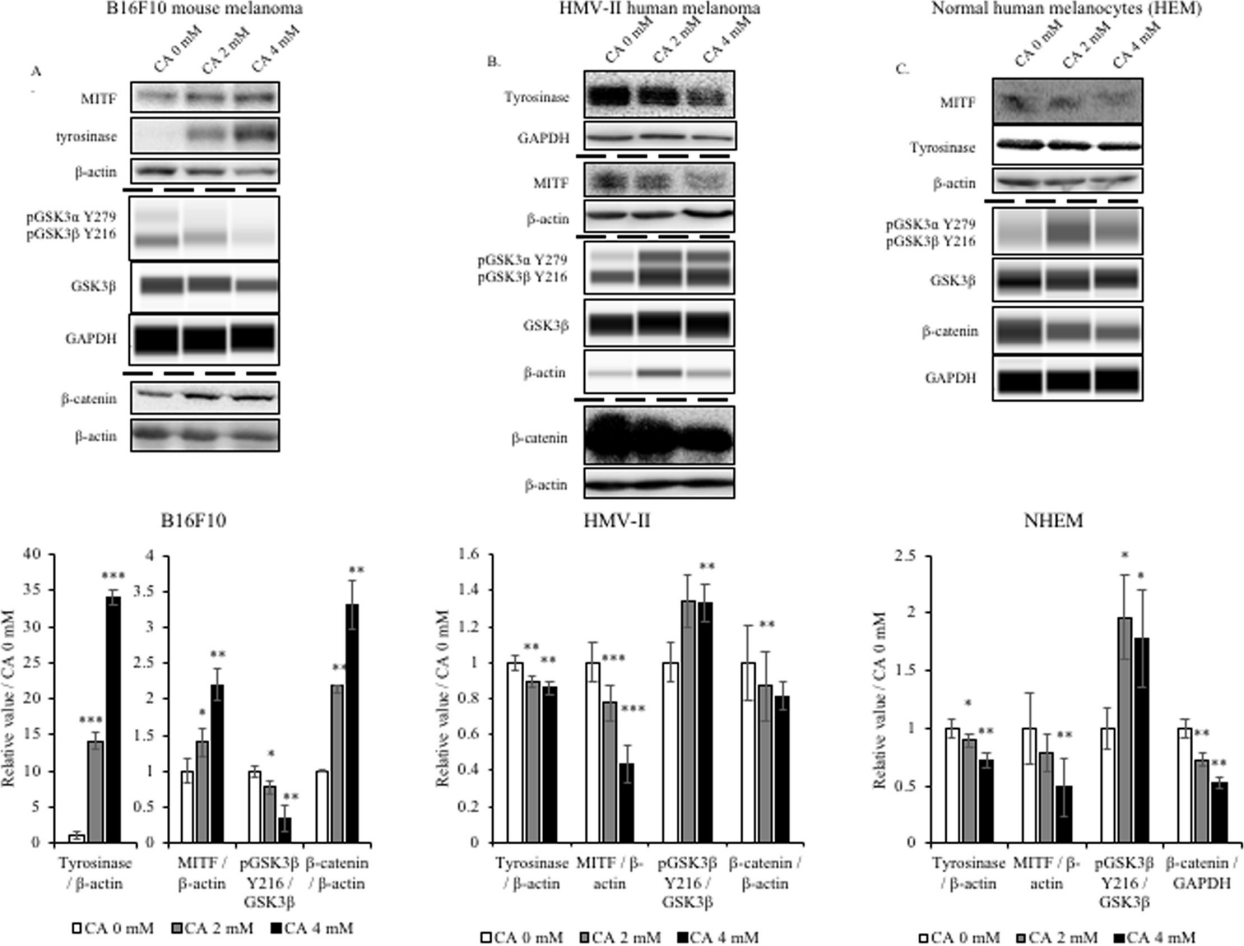
Effect of citric acid on the tyrosinase, MITF, and β-catenin protein levels in B16F10, HMV-II, and HEM cells. After treatment with citric acid (CA) (4 days for B16F10 and 6 days for HMV-II and HEM), cells were collected, and western blotting was performed to determine the expression of tyrosinase, MITF, and β-catenin in all three cell lines. (A, B, C) Protein levels detected using western blot analysis. Bar graphs represent the quantification of the western blot signals, analyzed using Image J. Results are presented as mean ± SD. Statistical analysis was performed using the Student’s t-test versus the non-treated groups (0 mM), n ≥ 3, *P < 0.05;**P < 0.01; ***P < 0.001.

First, we analyzed protein expression in B16F10 mouse melanoma cells. Consistent with the results of the melanin synthesis assay, citric acid treatment up-regulated the expression of tyrosinase and MITF (Fig 4A). According to the results of previous studies, β-catenin, as a transcription factor of MITF, regulates the expression of MITF, and thus the synthesis of melanin. Without the activation of the Wnt signaling pathway, GSK3β promotes the degradation of β-catenin, thereby inhibiting the expression of MITF and synthesis of melanin. Of note, GSK3β has two important phosphorylation sites, commonly known as the inactive Serine 9 (S9) and active tyrosine 216 (Y216). The active pGSK3β Y216 forms a complex with β-catenin, causing the phosphorylation of β-catenin and leading to its degradation [27]. Therefore, we assessed the levels of GSK3β and its phosphorylated form at the Y216 site. Results showed that citric acid treatment reduced the levels of p-GSK3β Y216 without changing GSK3β expression (Fig 4A). Afterward, we also assessed the intracellular protein level of β-catenin. Results showed that citric acid increased the β-catenin levels (Fig 4A).

Next, we conducted the same tests on the two human cell lines. In human melanoma cells (HMV-II), the expression of tyrosinase and MITF was reduced after treatment with citric acid, aligning with the intracellular melanin content of HMV-II cells (Fig 4B). β-Catenin expression was reduced, and the levels of p-GSK3β Y216 were increased (Fig 4B). Using the same method, we also detected the expression of melanin synthesis-related proteins in normal human epidermal melanocytes (NHEM). Consistent with the results of the melanin synthesis experiment, citric acid also reduced the expression of MITF and tyrosinase in NHEM cells (Fig 4C). The levels of p-GSK3β Y216 were also increased, and those of β-catenin decreased in NHEM cells (Fig 4C). Thus, we concluded that citric acid impacts tyrosinase activity and melanogenesis via the regulation of GSK3β and β-catenin; of note, the same concentration of citric acid exerts opposite effects on human and mouse cells.

### GSK3β is important in the citric acid-mediated regulation of melanogenesis

**Fig 5:**
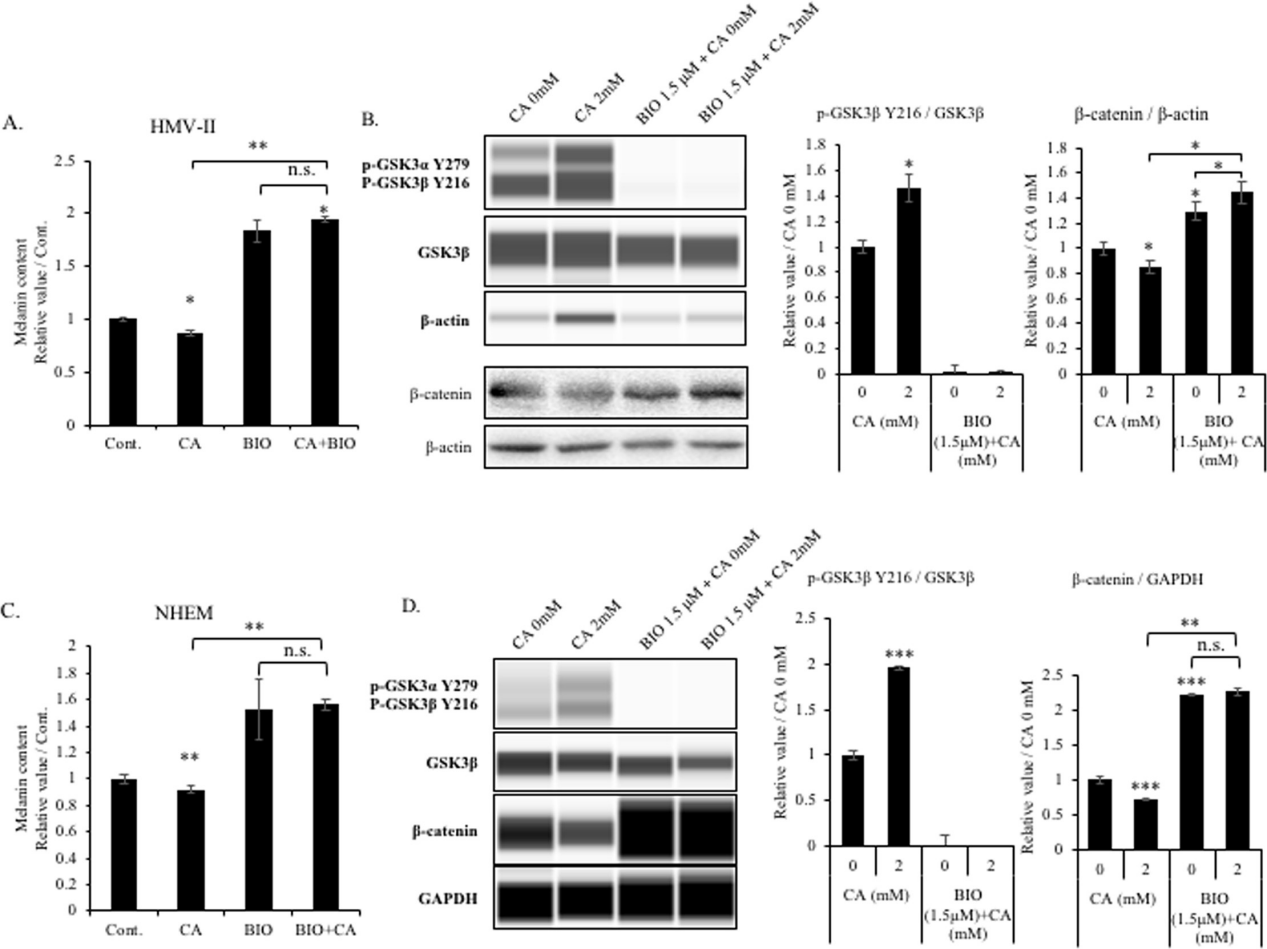
GSK3β is important for the citric acid-mediated regulation of melanogenesis. HMV-II cells and NHEMs were treated with or without citric acid (CA), BIO, or BIO+CA for 6 days. (A and C) The intracellular melanin content was measured to determine the effects of CA and BIO. CA and BIO decreased and increased the synthesis of melanin, respectively. CA did not affect the melanin content of BIO-treated cells. (B and D) The relative expression of p-GSK3β and β-catenin was detected using western blot analysis. CA increased the level of p-GSK3β and decreased that of β-catenin, while BIO showed the opposite effect. CA did not affect BIO treatment-induced protein expression. Bar graphs depict the quantitative results, which were analyzed using Image J. Results are presented as mean ± SD. Statistical analysis was performed using the Student’s t-test versus the non-treated groups (0 mM), n ≥ 3, *P < 0.05; **P < 0.01; ***P < 0.001; n.s. not significant.

To further investigate the role of GSK3β in the citric acid-mediated regulation of melanin synthesis, we used a GSK3β inhibitor (BIO) in experiments with HMV-II cells and NHEMs. After treatment with the GSK3β inhibitor, the intracellular melanin content was increased in line with our expectations, while the inhibitory effect of citric acid on melanin synthesis was blocked (Fig 5A and 5C). We also determined the expression of related proteins in the cells after BIO treatment. GSK3β phosphorylation increased after citric acid treatment and decreased after BIO treatment (Fig 5B and 5D). Furthermore, the β-catenin levels were also decreased in the context of citric acid treatment and increased in BIO-treated cells, opposing the effects observed in the context of GSK3β phosphorylation. Of note, in the presence of BIO, β-catenin levels were not impacted by citric acid treatment (Fig 5B and 5D). In summary, the comparison of melanin synthesis and the expression of signaling factors between citric acid-treated and citric acid + BIO treated-cells revealed that the regulatory effect of citric acid on melanin synthesis was completely blocked by the treatment with BIO. Therefore, we concluded that GSK3β is important for the citric acid-mediated regulation of melanin synthesis.

## Discussion

In this study, we mainly investigated and identified the mechanism via which citric acid regulates melanogenesis in B16F10 melanoma cells, HMV-II human melanoma cells, and normal human epidermal melanocytes (HEM). To the best of our knowledge, this is the first study to show that equal concentrations of citric acid exert opposite effects on melanin synthesis in human and mouse cells.

Previous studies have shown that citric acid can inhibit the proliferation of cells [28], such as normal human keratinocytes (HaCaT) [19] and various mouse or human cancer cells [23, 24]. We observed that a higher concentration of citric acid (6 mM) is sufficient to inhibit the proliferation of human and mouse cancer cells, which is consistent with the results of previous studies. We also assessed the expression of cell cycle-specific proteins (cyclin D1 and E1), which supported the cell proliferation inhibitory function of citric acid (S1 Fig in S1 File). Here, we observed that the effects of citric acid on the proliferation of human and mouse cells were identical. Previous studies have reported the apoptosis-inducing effect of citric acid [19, 24, 28, 29], and suggested that citric acid can act as a candidate anti-tumor agent. We also tested the effect of citric acid on apoptosis by detecting caspase-3 activity using an apoptosis detection kit. Interestingly, we observed that citric acid promoted apoptosis in mouse cancer cells, but did not induce apoptosis in normal human epidermal melanocytes (S2 Fig in S1 File). This indicates that citric acid might affect mouse and human cells differently.

The melanin in the epidermis is generally considered to protect against UVR-induced damage. The oxidative stress in UV-stimulated epidermal keratinocytes may cause genetic mutations or may photodamage DNA. To avoid this, keratinocytes secrete the α-melanocyte-stimulating hormone (α-MSH) to promote the secretion of melanin by the surrounding melanocytes, thereby preventing the damage caused by UV radiation [2, 4, 5, 9]. Our results indicated that citric acid did not directly regulate the redox reaction; as compared to ascorbic acid (vitamin C) and BHT, citric acid showed almost no antioxidant capacity in the DPPH assay. However, citric acid added to the cells regulated the intracellular redox reaction. In agreement with the results of other studies [18, 30], this indicated that citric acid is an auxiliary agent assisting in the progression of the redox reaction. Used as an additive, citric acid is believed to reduce oxidative stress [18, 24], which is also consistent with the effect of citric acid on human cells; however, we observed the opposite effect in mouse cells.

The UV-induced increase in ROS promotes the synthesis of melanin, indicating that ROS plays an important role in the regulation of melanin synthesis [4]. The increase and decrease in intracellular ROS levels were consistent with the increase and decrease in melanin content in mouse and human cells, respectively. However, other studies have suggested that melanin synthesis can also promote ROS generation [2, 31–33], and that an increase in ROS induces MITF degradation and decreases melanin synthesis via the MAPK/ERK signaling pathway [34–36]. Although these results appear contradictory, they warrant further investigations on the relationship between ROS generation and melanin synthesis.

Tyrosinase is an important rate-limiting enzyme that regulates melanin synthesis; the expression of tyrosinase is regulated by the transcription factor, MITF [7–9]. Our results showed that citric acid was able to regulate the levels of these two proteins, which was consistent with the results of melanin synthesis and tyrosinase activity assays.

Adding citric acid directly to the culture medium will cause a change in the pH. According to previous studies [37], lower pH (e.g. in the range of 3 to 5) will greatly reduce the catalytic activity of tyrosinase and cause a decrease in the ability of melanosomes to synthesize melanin. In this experiment, the direct addition of citric acid to the culture medium led to a diminishment of the pH from the original 7.2–7.3 to 7.1–7.2 (in DermalLife base medium) and to 7.0–7.1 (in RPMI-1640); of note, the original pH was restored within 24 h. Therefore, we believe that although the concentration of 2–4 mM citric acid seems to be a little bit high, it is not enough to significantly reduce the pH of the medium, due to the presence of buffers such as NaHCO_3_.

To investigate the upstream events in the context of the citric acid-mediated regulation of melanin synthesis, we studied the signaling pathway that controls MITF, a critical transcription factor. First, under normal circumstances (in the epidermis), ROS generated by UV irradiation promotes the secretion of α-MSH by keratinocytes, which then activates the cAMP-PKA-CREB signaling pathway via the MC1R receptors on the membrane of melanocytes to promote MITF gene expression [7–9]. However, we did not use α-MSH as a positive control in this study, as we observed 1) that the relationship between ROS and melanin synthesis is complex and 2) that although the MAPK/ERK pathway participates in the regulation of melanin synthesis, this pathway did not play a central role after pyruvic acid treatment [21]; hence, we focused on the signaling pathways related to GSK3β in this study.

β-Catenin (a multifunctional protein) is an important downstream effector in the Wnt signaling pathway and can act as a transcriptional coactivator via the interaction of the T-cell transcription factor (TCF)/lymphoid enhancer-binding factor (LEF) family of DNA binding proteins in the nucleus [13]. In the absence of the Wnt signal, GSK3β, together with axin and adenomatous polyposis coli protein (APC), forms a complex that phosphorylates β-catenin, inducing β-catenin degradation via the proteasome. In the presence of Wnt signaling, the GSK3β-dependent inhibition of β-catenin is blocked. β-catenin and LEF1 together bind to the MITF promoter via the binding site on LEF-1 and promote MITF expression [13, 14, 38, 39]. Our results showed that β-catenin expression increased in mouse cells and decreased in human cells after citric acid treatment, which was consistent with the MITF expression and GSK3β phosphorylation levels. Furthermore, the inhibition of GSK3β (using BIO) prevented the degradation of β-catenin; when GSK3β was blocked, citric acid did not affect the expression of β-catenin. Although BIO is a highly effective GSK3 inhibitor, it is also a pan-JAK inhibitor. To exclude the non-specificity of BIO, using a method that specifically allows the reduction of GSK3β activity without impacting the expression of GSK3 will be further insightful to prove the importance of GSK3β.

MITF has three recognized phosphorylation sites: Ser73 (phosphorylated by ERK) [34], and Ser298 and Ser409 (phosphorylated by GSK3β). MITF phosphorylated at Ser73 and Ser409 has been shown to be degraded via the ubiquitin-proteasome system [15, 40]. The GSK3β-mediated phosphorylation at Ser298 increases the binding activity of MITF; of note, this is a feature of the Waardenburg syndrome type II, a rare genetic disease characterized by pigmentation defects [17, 40]. Taken together, GSK3β can directly or indirectly regulate MITF activity to control melanin synthesis. The inhibitory effect of GSK3β on melanin synthesis was also demonstrated in this study (Fig 5).

The mouse melanoma cancer cell B16F10 is widely used as an experimental cell line in many studies on melanin synthesis [9, 12, 34, 35, 39, 41]. Therefore, in the initial stage of this study, we used the same cell line for experiments. However, we obtained opposite results when we used human melanocytes, which we also used in previous studies. Therefore, we used normal human epidermal melanocytes (NHEM) and HMV-II human melanoma cells to investigate the regulatory mechanism of citric acid. Although we have delineated the mechanism via which citric acid regulates melanin synthesis, we cannot explain why citric acid exerts different effects on mouse and human cells. Therefore, we speculate the following based on relevant literature. First, many studies have shown that citric acid possesses anti-cancer activity in various cells [22–24, 29, 42]. In particular, cancer cells tend to depend on glycolysis for energy production, rather than on the TCA cycle, the most efficient energy production pathway [42]. However, as an important metabolic process, the TCA cycle is not absent in cancer cells [20]. Previous studies have shown that glycolysis and the TCA cycle are repressed after cells are treated with exogenous citric acid, which suppresses tumor growth [24, 29]. Second, citric acid can promote cancer cell apoptosis [19, 23, 29, 43], which is related to the mitochondrial metabolic balance [44]. Therefore, these findings suggest that citric acid-mediated induction of apoptosis occurs due to the effect of exogenous citric acid on mitochondrial homeostasis [19, 23, 29, 43]. Based on these previous results, we further speculate that treatment with exogenous citric acid might affect mitochondrial homeostasis, thereby disrupting the balance between energy and nutrient metabolism via glycolysis and the TCA cycle, subsequently affecting the intracellular ROS metabolism. It is well known that the tolerance and homeostasis of human and mouse cells vary; hence, the extent of the intracellular metabolic balance disruption caused by the same concentration of citric acid may differ, resulting in the differential effect of citric acid on melanogenesis in mouse and human cells. The case of citric acid (another example of the marked differences between the pre-clinical and clinical contexts) should also be remembered during the development of new drugs in the future.

Since both humans and mice are mammals, which share many common genetic features, mice and their cultured cells are usually used instead of humans for biological experiments. In the previous reports, it was also found that many reagents have similar effects on mice and humans. However, our results show that citric acid has an opposite regulatory effect on melanin synthesis in mouse cells and human cells. This raises a critical question, why and how citric acid differentially regulates melanin synthesis in mouse and human cells. Firstly, on average, the protein-coding regions of the mouse and human genomes are 85 percent identical; while the non-coding regions are less 50 percent similar [45]. Due to our ignorance of the non-coding regions of genes, there will be some differences in the process of mouse and human gene expression and regulation that we have not yet known, so it is still difficult to link the gene and phenomenon (like melanogenesis) [46]. Secondly, our results revealed that citric acid regulated the synthesis of melanin by regulating the activity of GSK3β. GSK3β (glycogen synthesis kinase-3beta) is a key regulatory enzyme in glucose metabolism [47], its activity is also related to the activity of mitochondria [48]. Previous studies have shown that the addition of citric acid will promote the production of ATP and inhibit the process of glycolysis [24]. Therefore, we hypothesize that citric acid will interact with mitochondria to achieve the effect of regulating GSK3β. Due to the difference in the tolerance of human cells and mouse cells to citric acid, this opposite regulation effect occurs. However, there is almost no relevant research on the relationship between citric acid and GSK3β before. Therefore, further research is necessary to explain and verify this idea.

Although we identified the differential effects of citric acid on human and mouse cell melanogenesis, we have not addressed the differences in metabolism between normal and cancer cells [49]. The upstream factors (GSK3β/β-catenin/MITF) that control melanin synthesis also control the expression of various downstream regulators of other cellular functions [38, 50, 51]. Therefore, although we did not observe any difference between normal (HEM) and cancer cells (HMV-II) regarding melanogenesis, we still expect that citric acid will act differently on normal and cancer cells in the regulation of other functions.

This study has limitations. First, it is limited to cell-based experiments; citric acid was directly added to the medium to act on each cell. However, we were unable to determine 1) whether the administration of citric acid to mice will show the same results; 2) whether citric acid can be completely absorbed; 3) whether the method of administration (gavage, subcutaneous injection, mixed feeding in water, etc.) can influence the effect of citric acid on melanogenesis. We aim to investigate these points in the future. Second, we only used three cell lines (B16F10, HMV-II, and NHEM) to verify the mechanism behind the citric acid-mediated regulation of melanogenesis in human and mouse cells in this study, but did not investigate the role of other cells in this process. In the future, we will focus on other cell types involved in pigmentation, such as adipocytes, muscle cells, and immune cells. Third, there is an increasing indication that interspecific phenotypic differences result from variations in gene-regulatory interactions [52, 53]. For example, in zebrafish, SOX10, MITF, and tyrosinase present a simple linear regulation relationship. However, it is more complicated in the mouse, which the SOX10 can also directly regulate the expression of tyrosinase [54]. In our results, we confirmed the simple linear regulation of GSK3β and β-catenin on MITF and further tyrosinase. But melanogenesis is a very complicated process, many other regulatory signaling pathways (KIT, EDNRB, Notch) for MITF are also included. While we only investigated the different regulatory effects of citric acid on GSK3β in this experiment. Therefore, it is interesting and meaningful to further investigate the species-specific regulatory effect by citric acid on other MITF related factors.

## Conclusions

In this study, we investigated the effect of citric acid on human and mouse melanogenesis. Citric acid increased melanogenesis in B16F10 mouse melanoma cells but decreased it in HMV-II human melanoma cells/human epidermal melanocytes (HEM). Moreover, we showed that citric acid influences melanogenesis via the regulation of the GSK3β and β-catenin signaling pathways. These results reveal a potential role of citric acid as a cosmetic effector, and highlight the differential effects of reagents on human and mouse cells.

## Supporting information

S1 File (PDF)

S1 Raw data (PDF)
