# Supplementary material for: Citric acid promoted melanin synthesis in B16F10 mouse melanoma cells, but inhibited it in human epidermal melanocytes and HMV-II melanoma cells via the GSK3β/β-catenin signaling pathway
Source: PLoS One. 2020 Dec 17;15(12):e0243565. doi: 10.1371/journal.pone.0243565 (PMC7746170; doi:10.1371/journal.pone.0243565)

S1 Fig Citric acid effect on cell cycle-related gene expression.

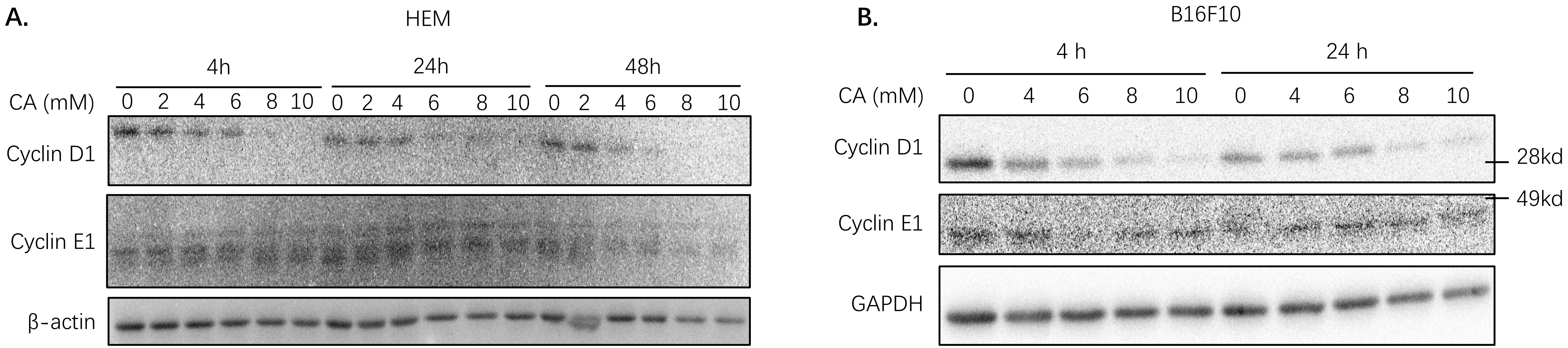

S2 Fig Citric acid effect on caspase-3 activity

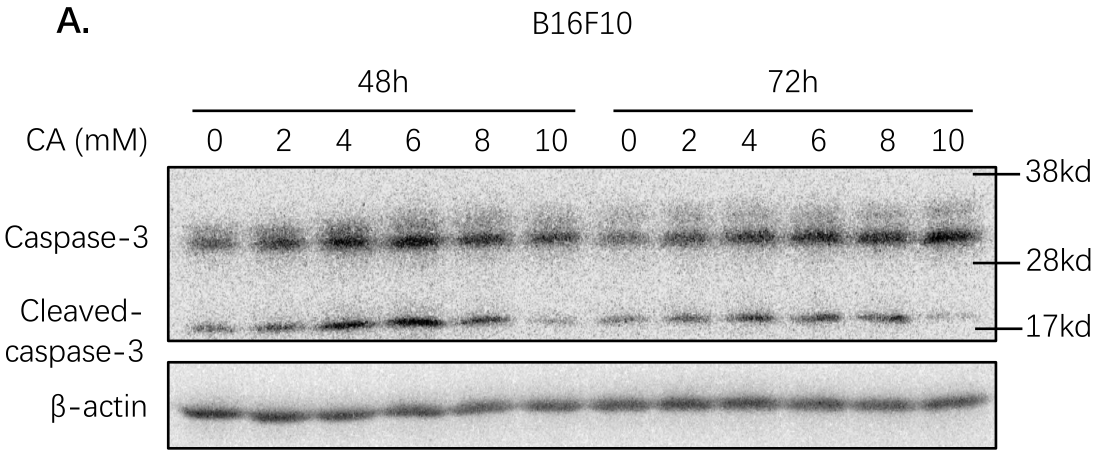

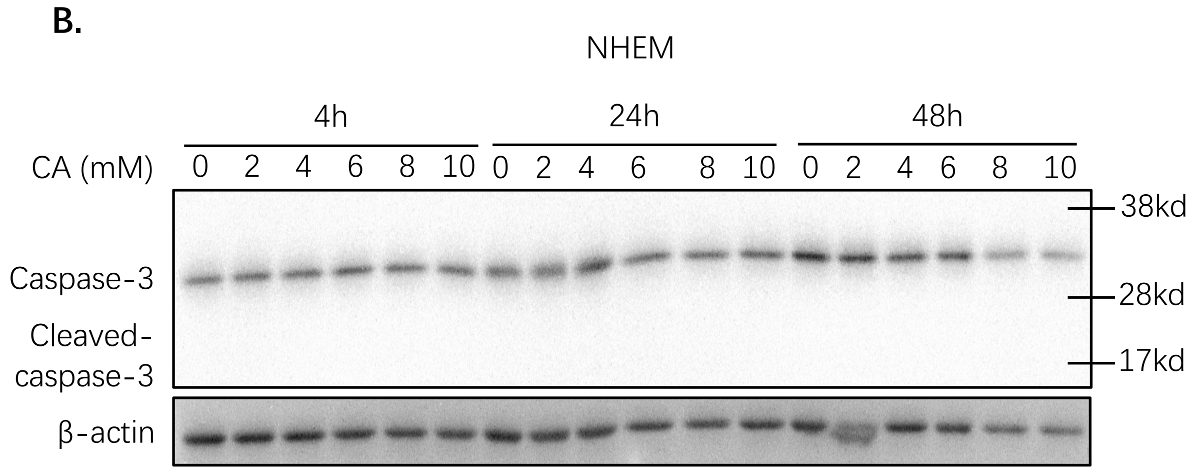

S3 Fig The BIO effect on HEM cells.

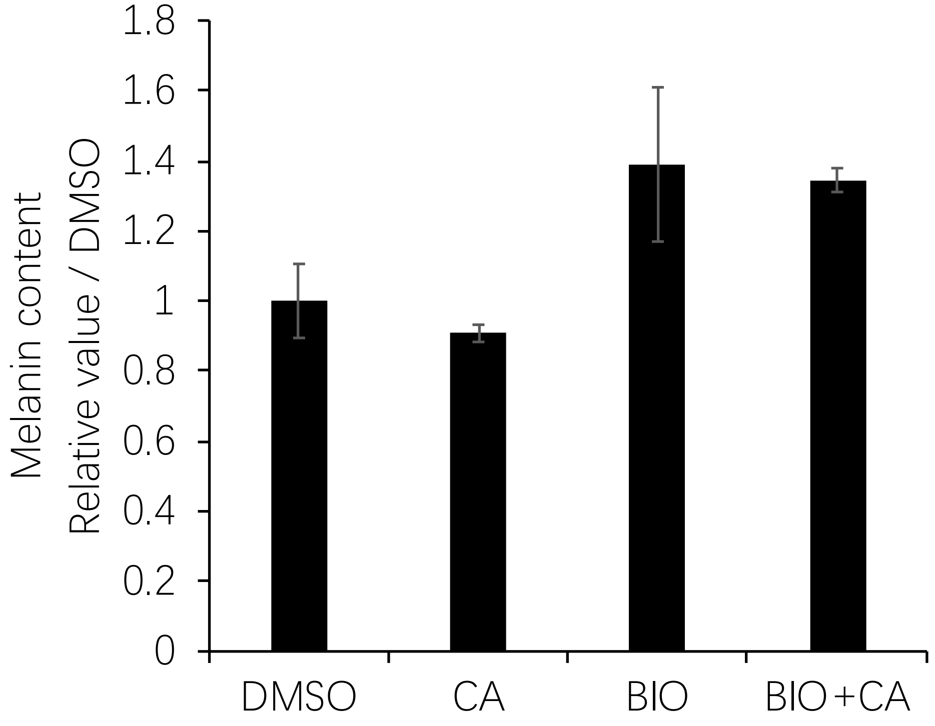

Supplement: S1 File — (PDF) [file pone.0243565.s001.pdf]
