# Supplementary material for: Citric acid promoted melanin synthesis in B16F10 mouse melanoma cells, but inhibited it in human epidermal melanocytes and HMV-II melanoma cells via the GSK3β/β-catenin signaling pathway
Source: PLoS One. 2020 Dec 17;15(12):e0243565. doi: 10.1371/journal.pone.0243565 (PMC7746170; doi:10.1371/journal.pone.0243565)
Supplement: S1 Raw data — (PDF) [file pone.0243565.s002.pdf]

Fig 4A Tyrosinase

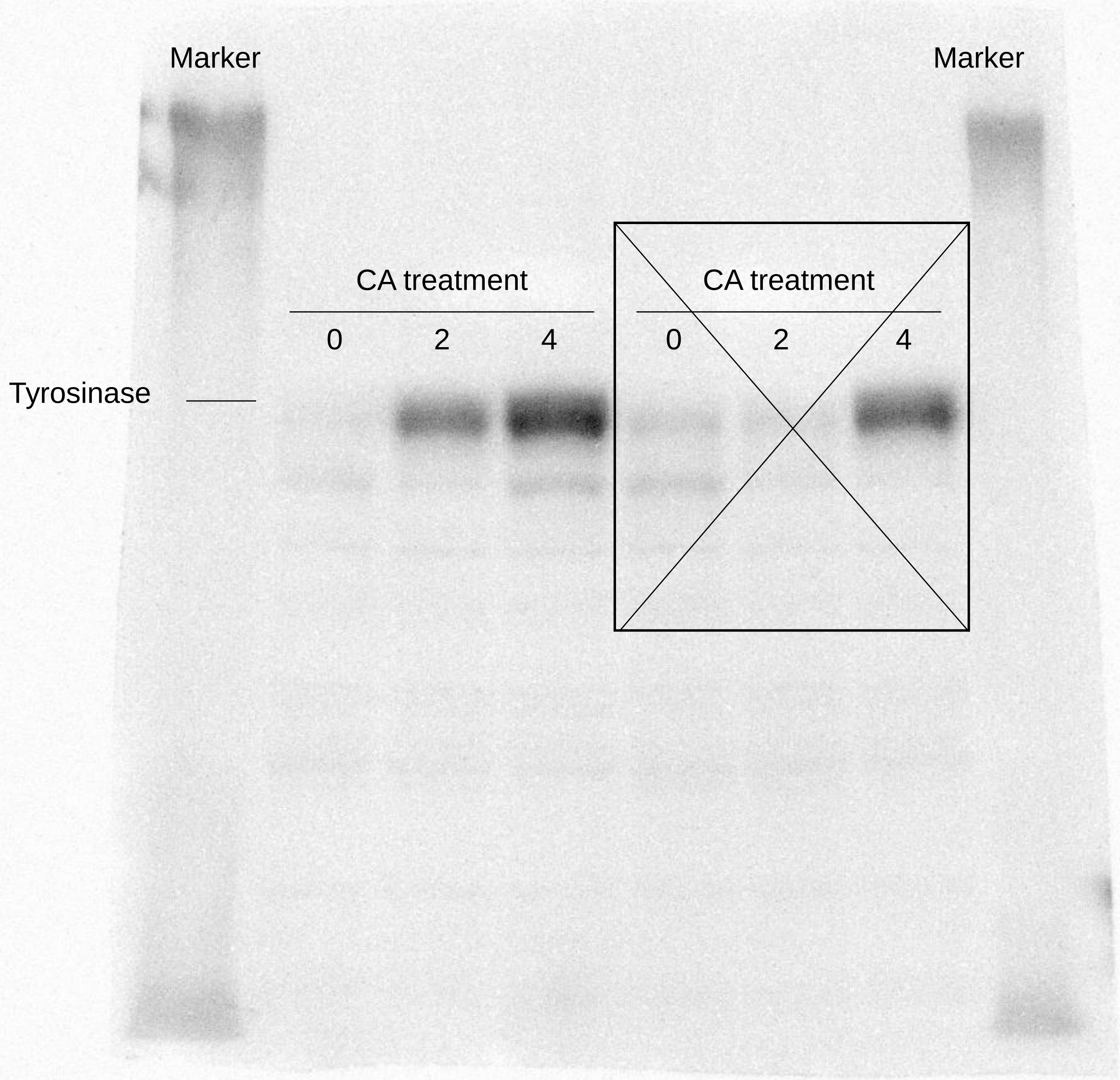

Fig 4A MITF

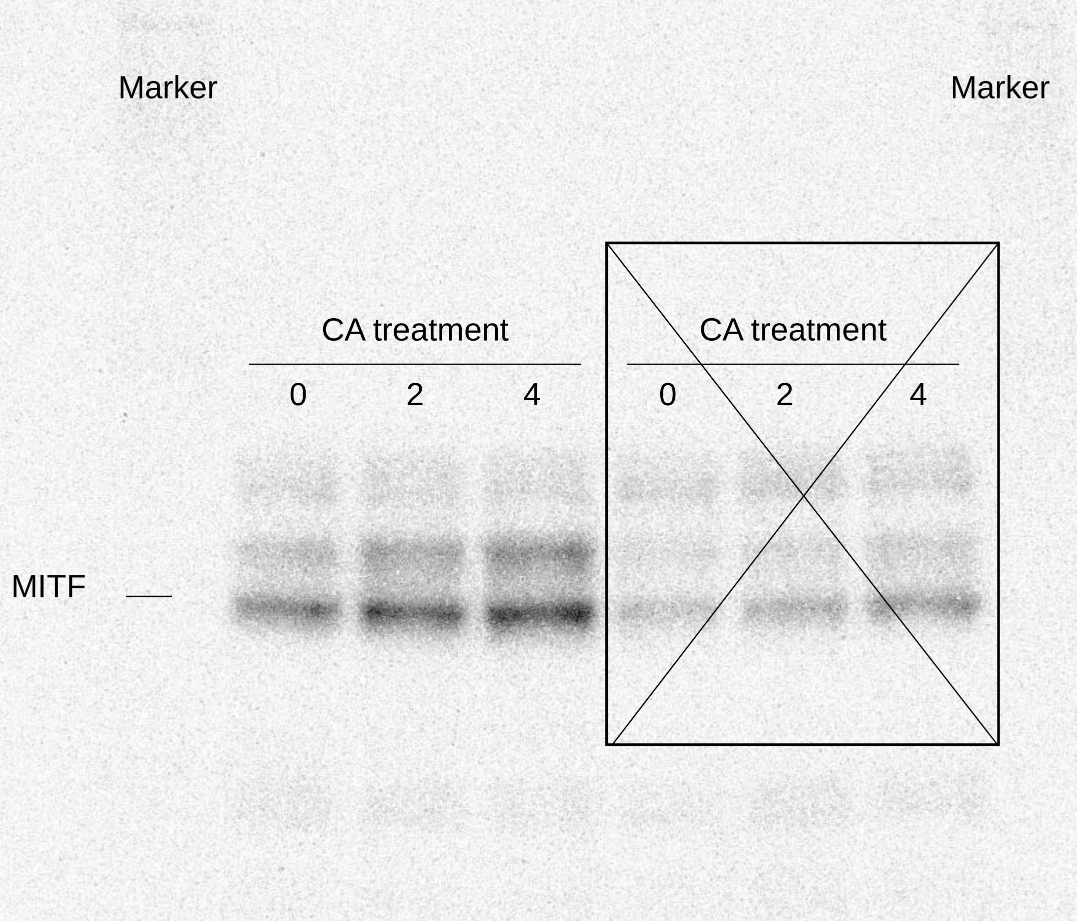

Fig 4A β-actin for TYR and MITF

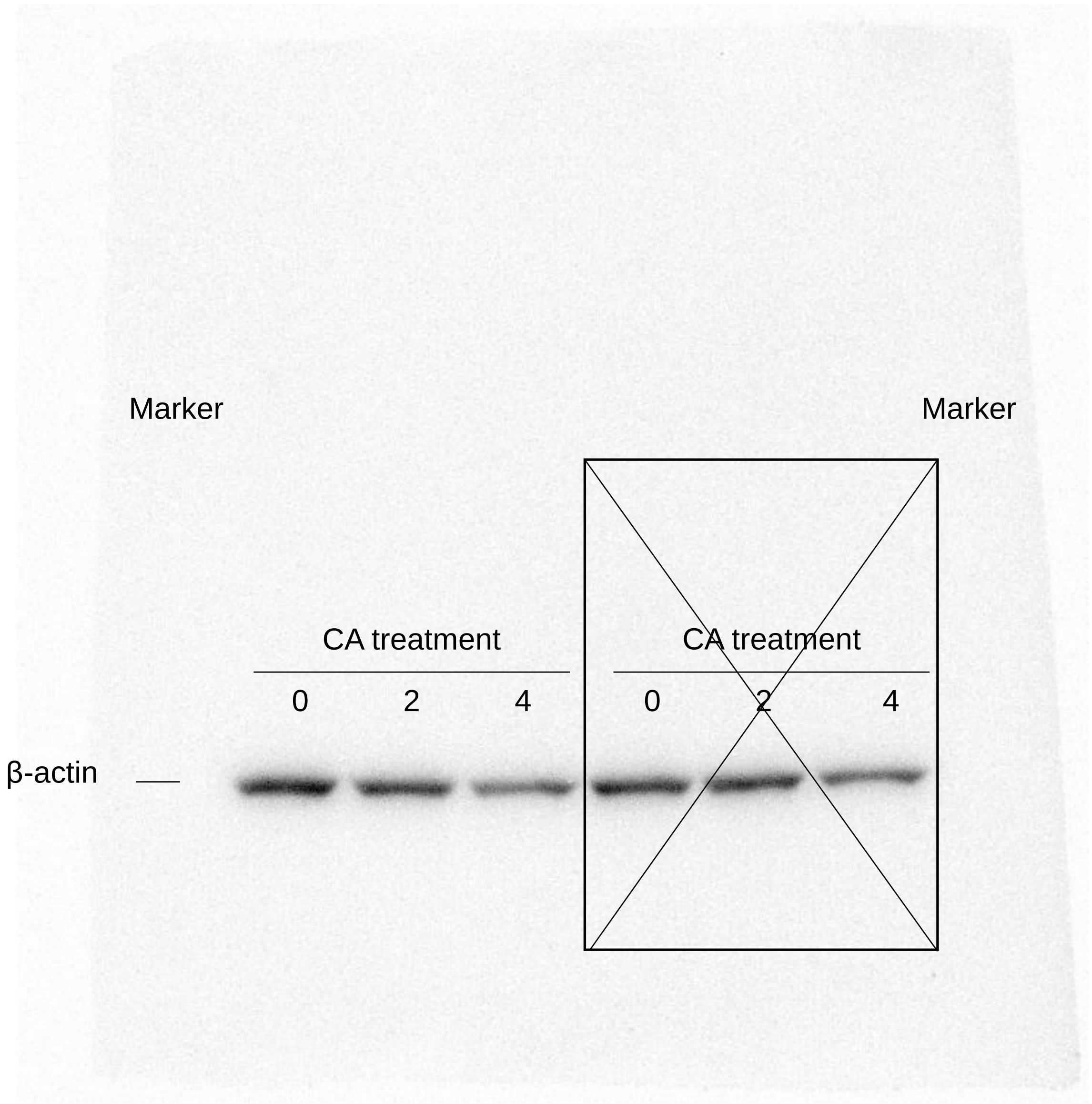

Fig 4A GSK3β, pGSK3β

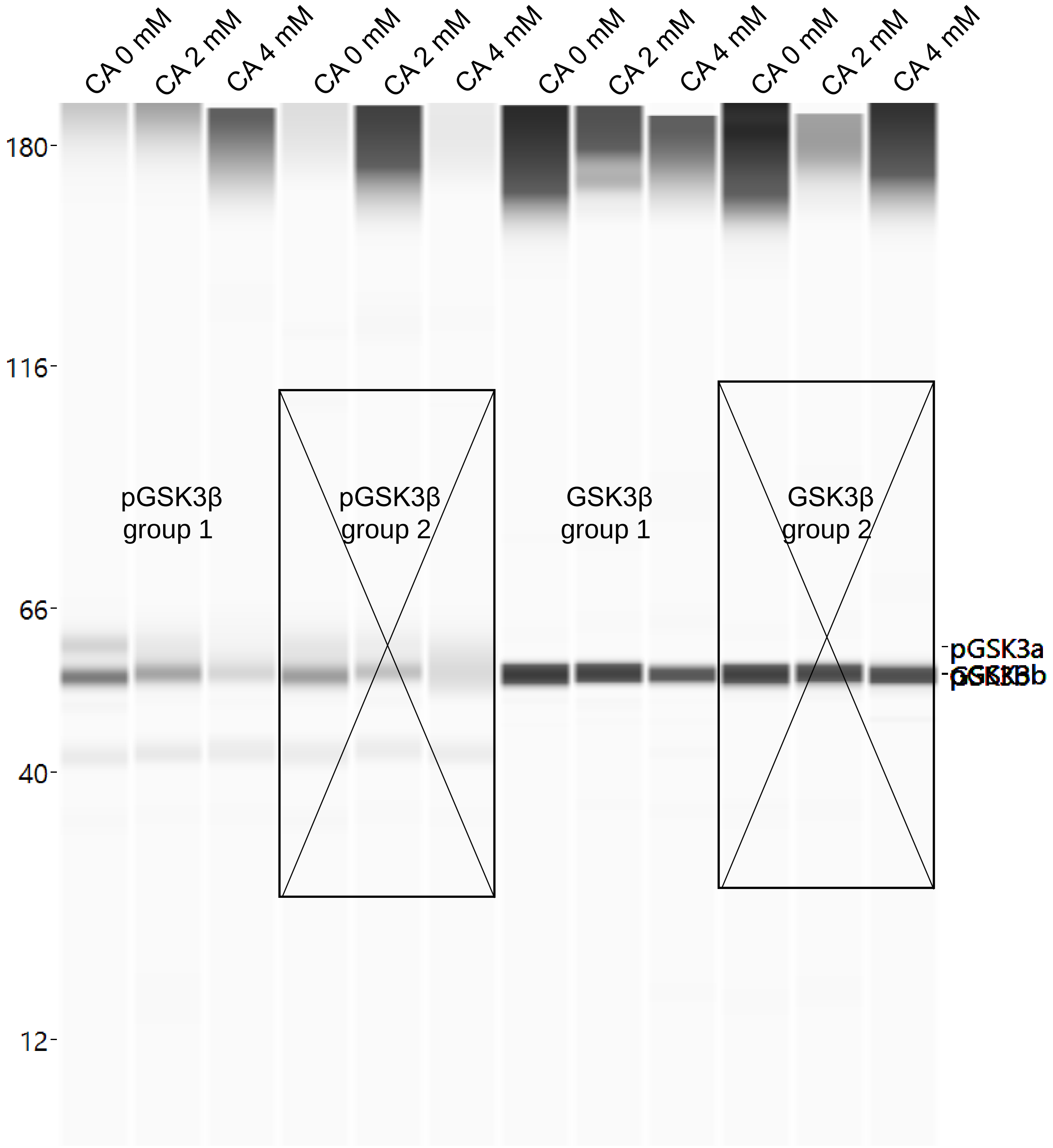

Fig 4A GAPDH

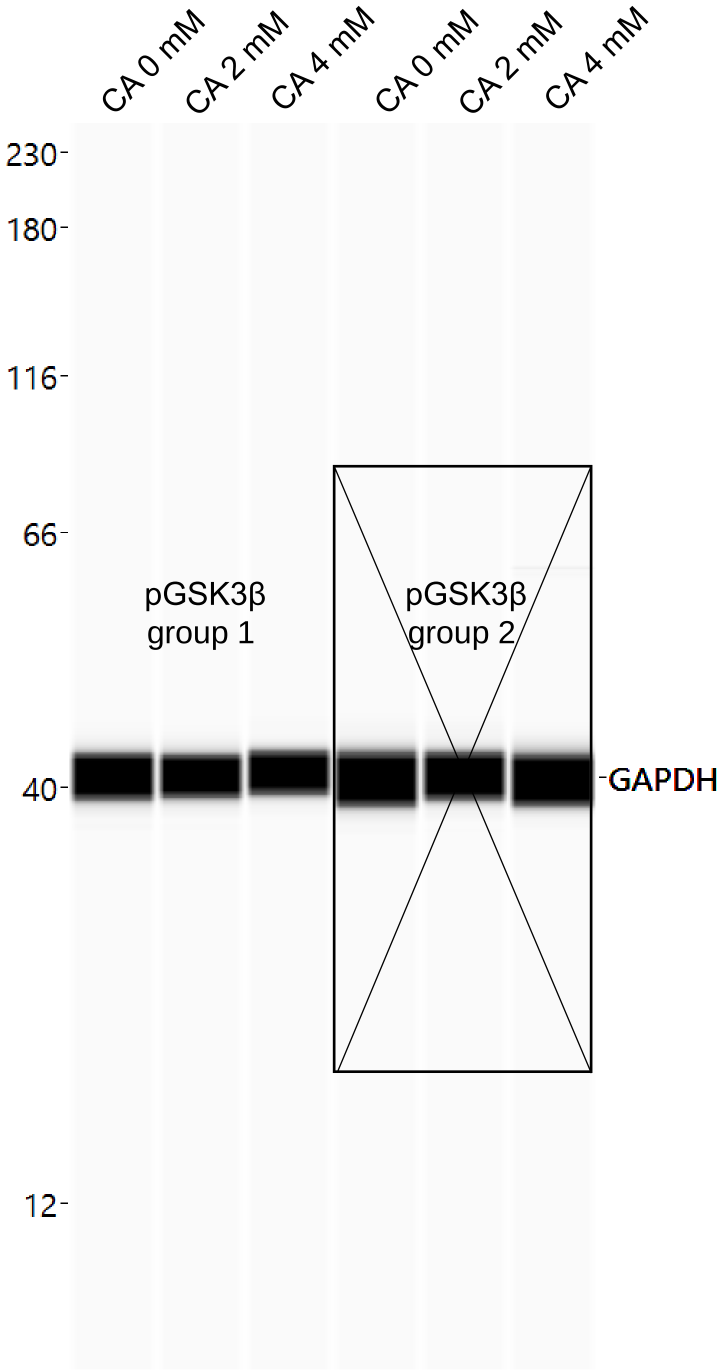
CA 0 mM
CA 2 mM
CA 4 mM
CA 0 mM
CA 2 mM
CA 4 mM
230
180
116
66
40
12
pGSK3β
group 1
pGSK3β
group 2
GAPDH

Fig 4A β-catenin

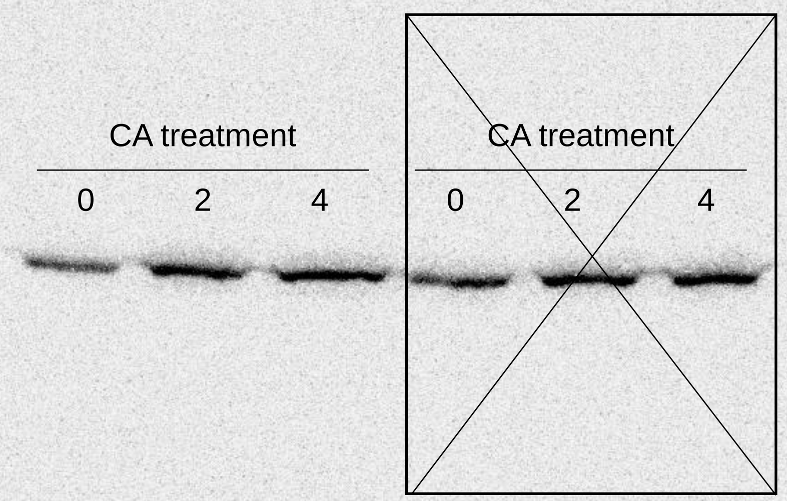

Fig 4A β-actin (β-catenin)

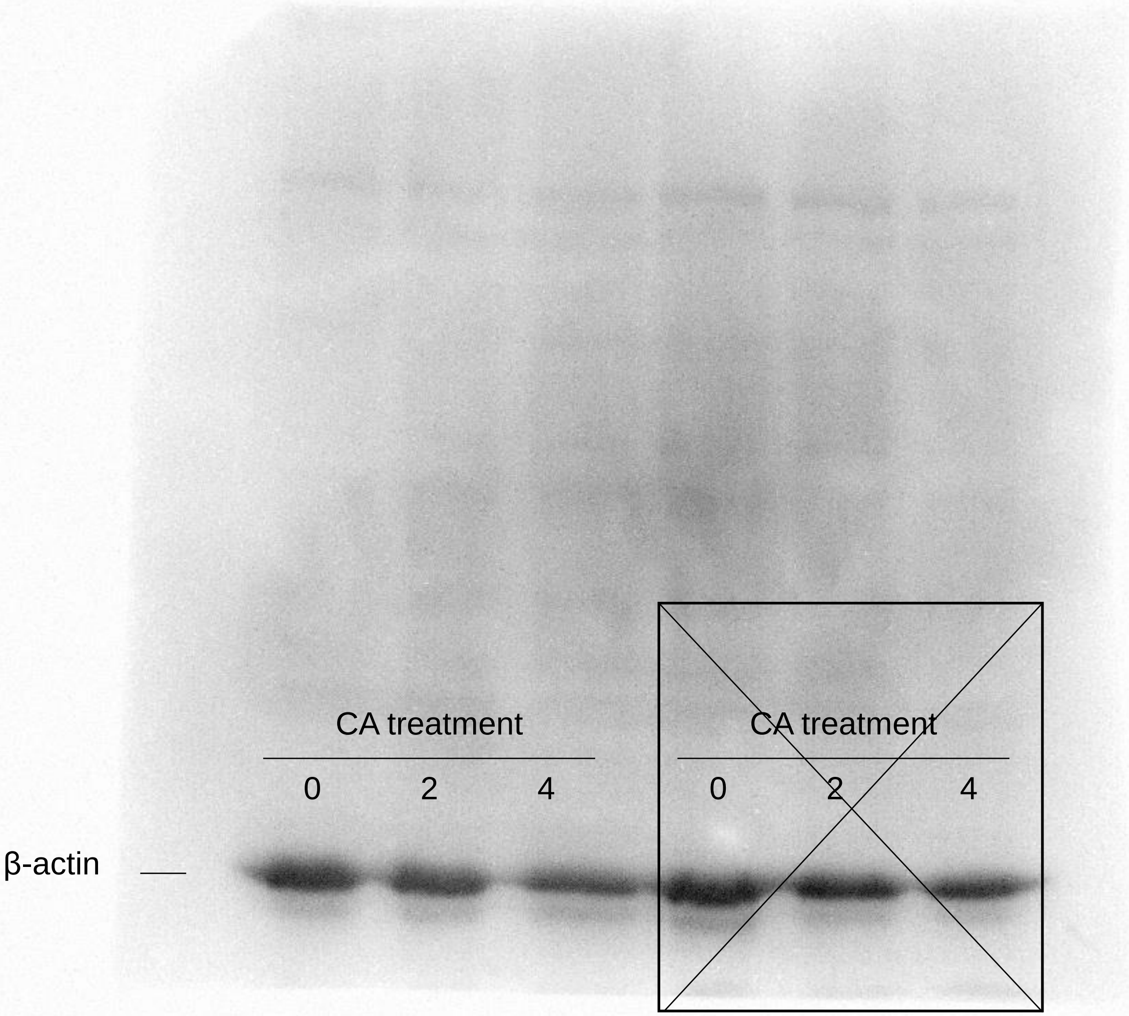

**Fig 4B MITF**

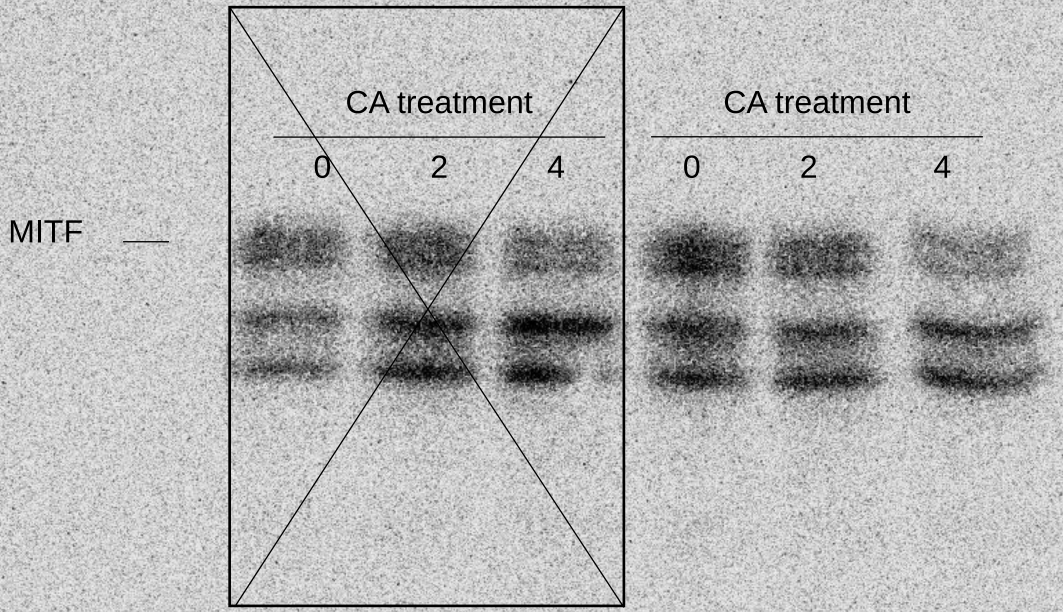

Fig 4B β-actin (MITF)

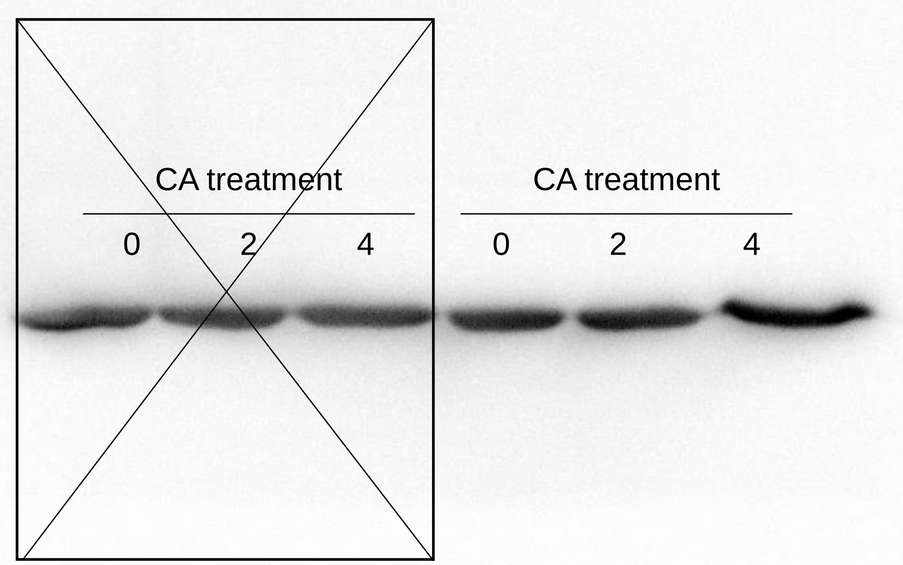

Fig 4B Tyrosinase

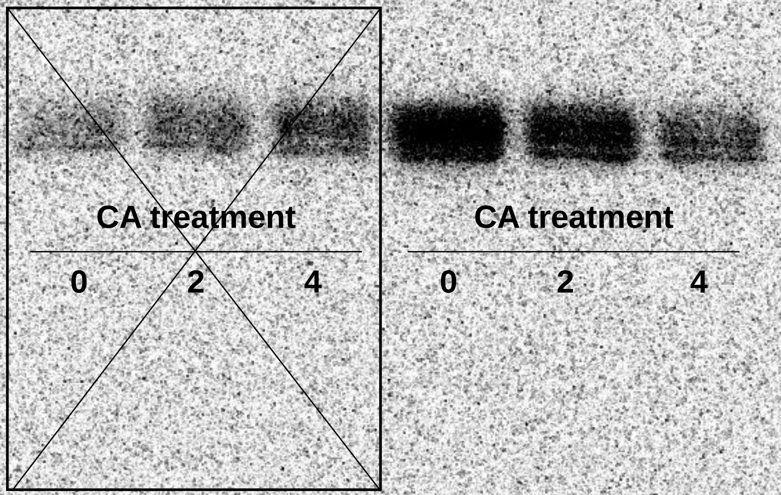

Fig 4B GAPDH (Tyrosinase)

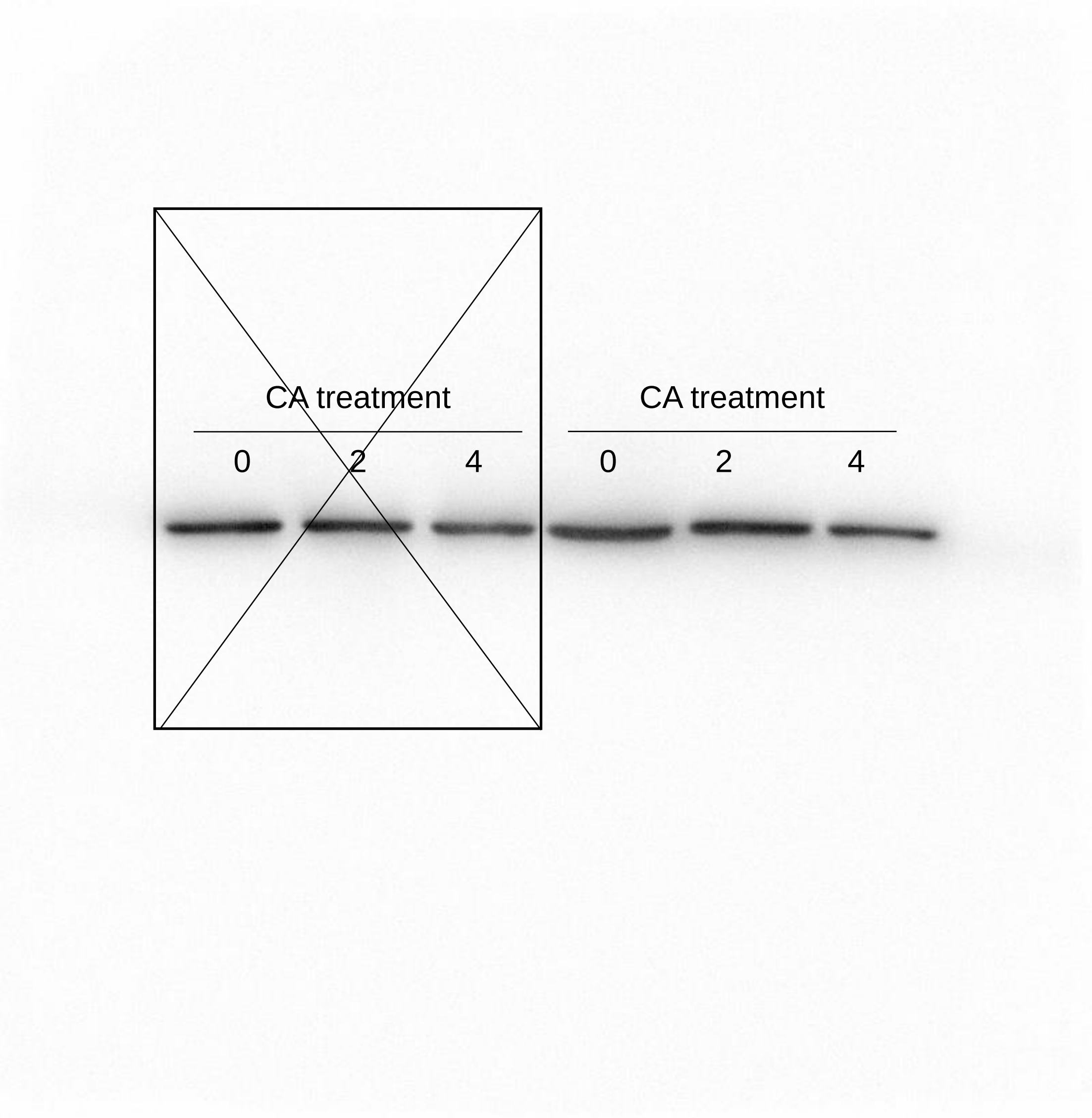

Fig 4B GSK3β, pGSK3β and β-actin

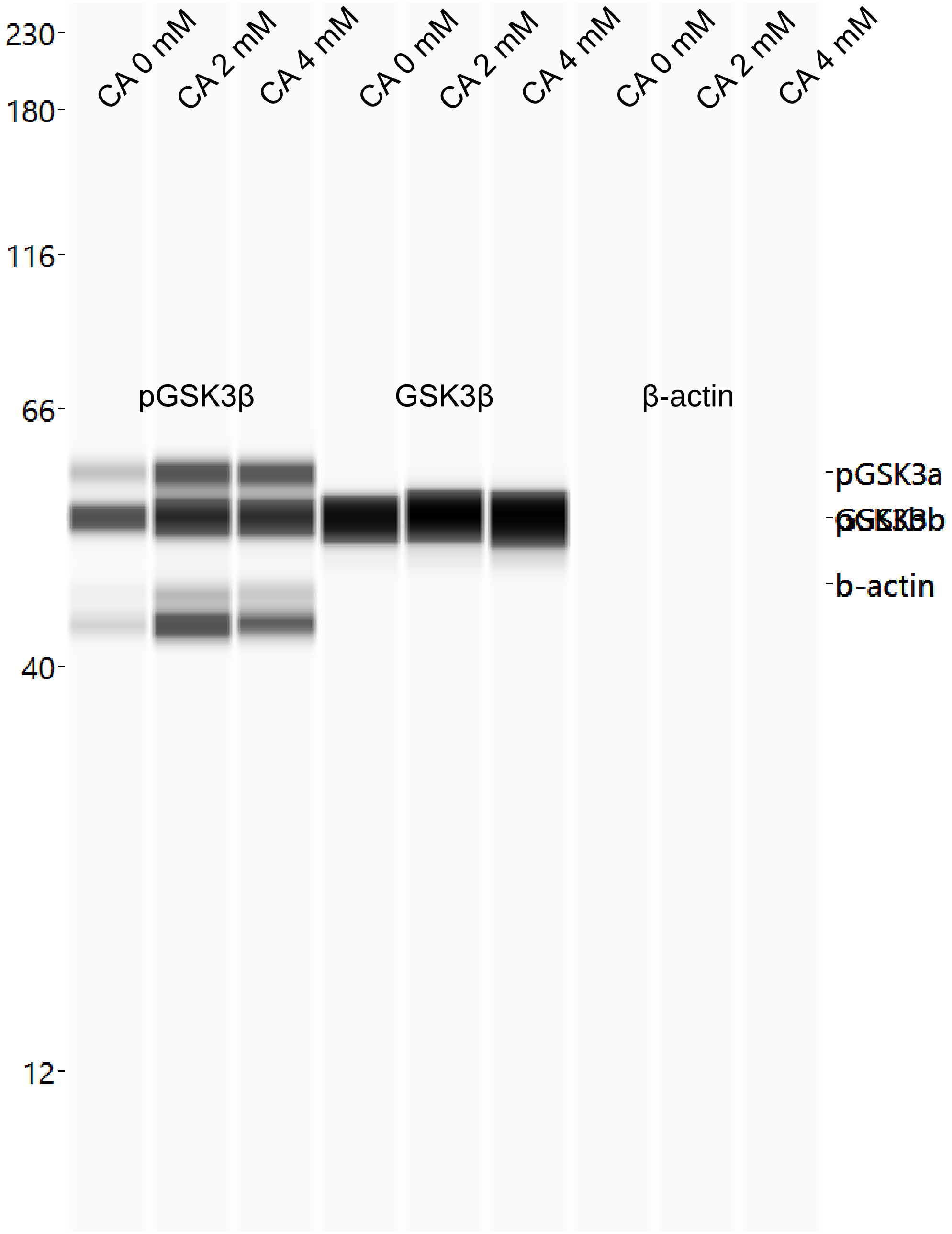

Fig 4B GSK3β, pGSK3β and β-actin (Contrast adjustment for β-actin)

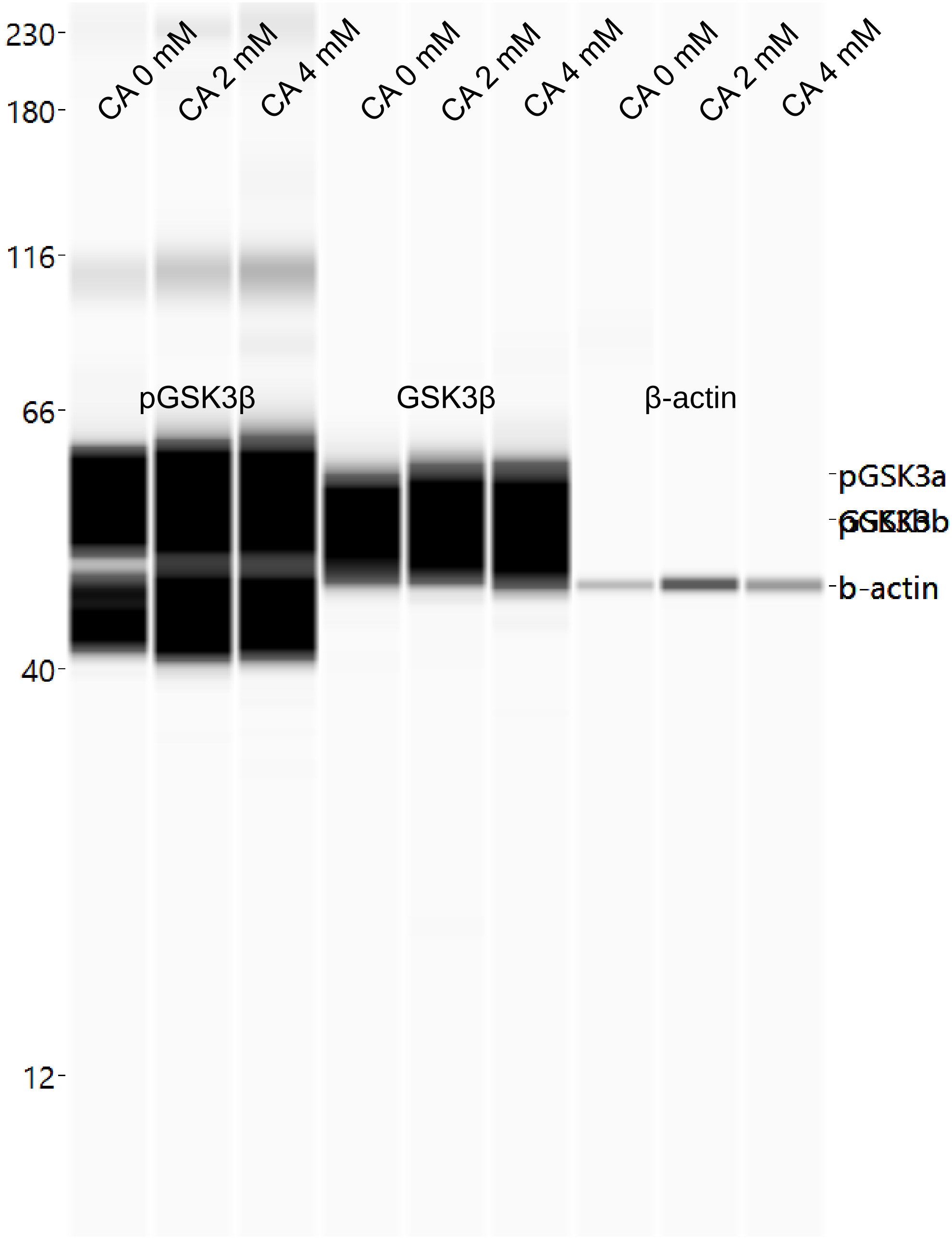

Fig 4B β-catenin

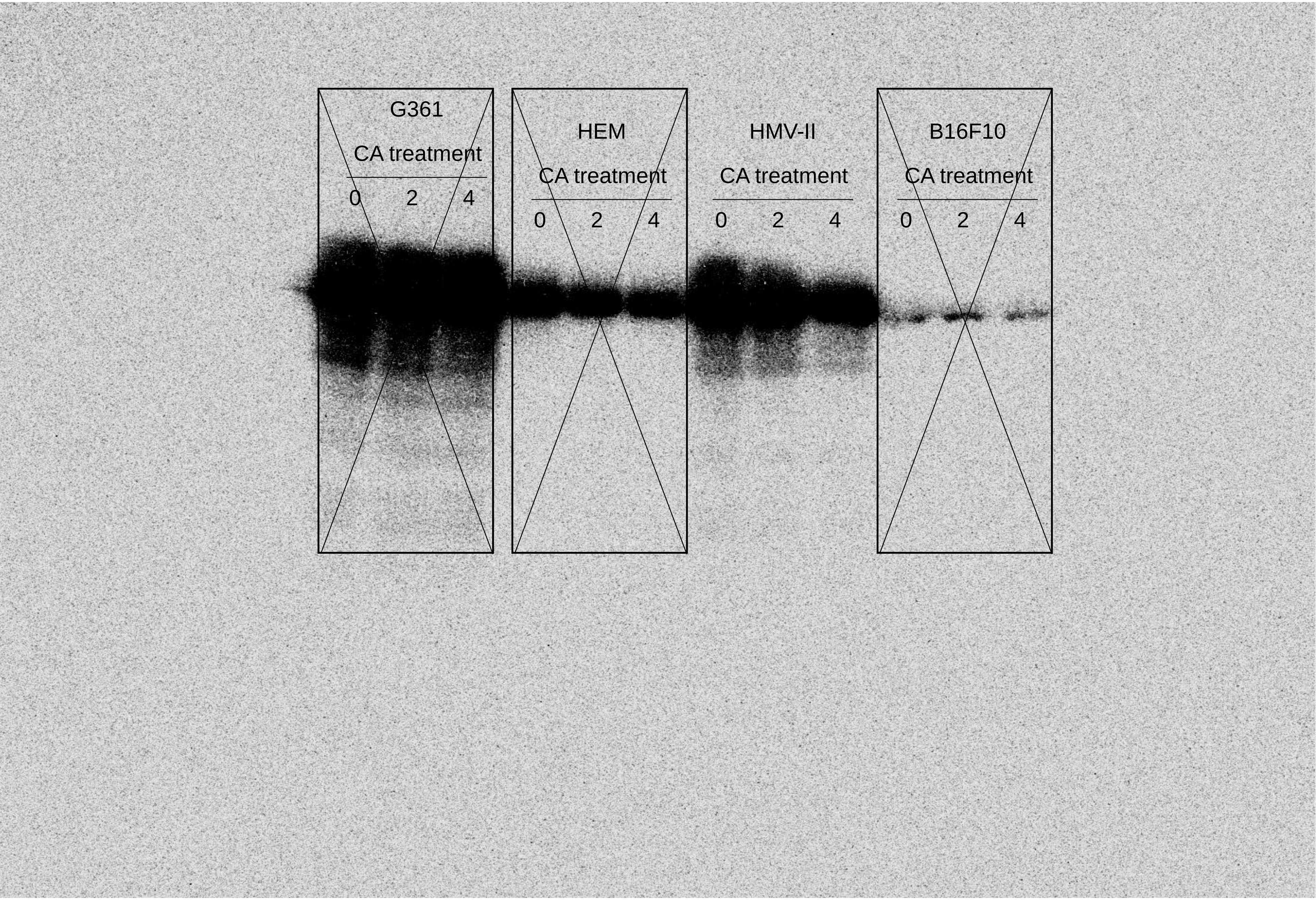

Fig 4B β-actin

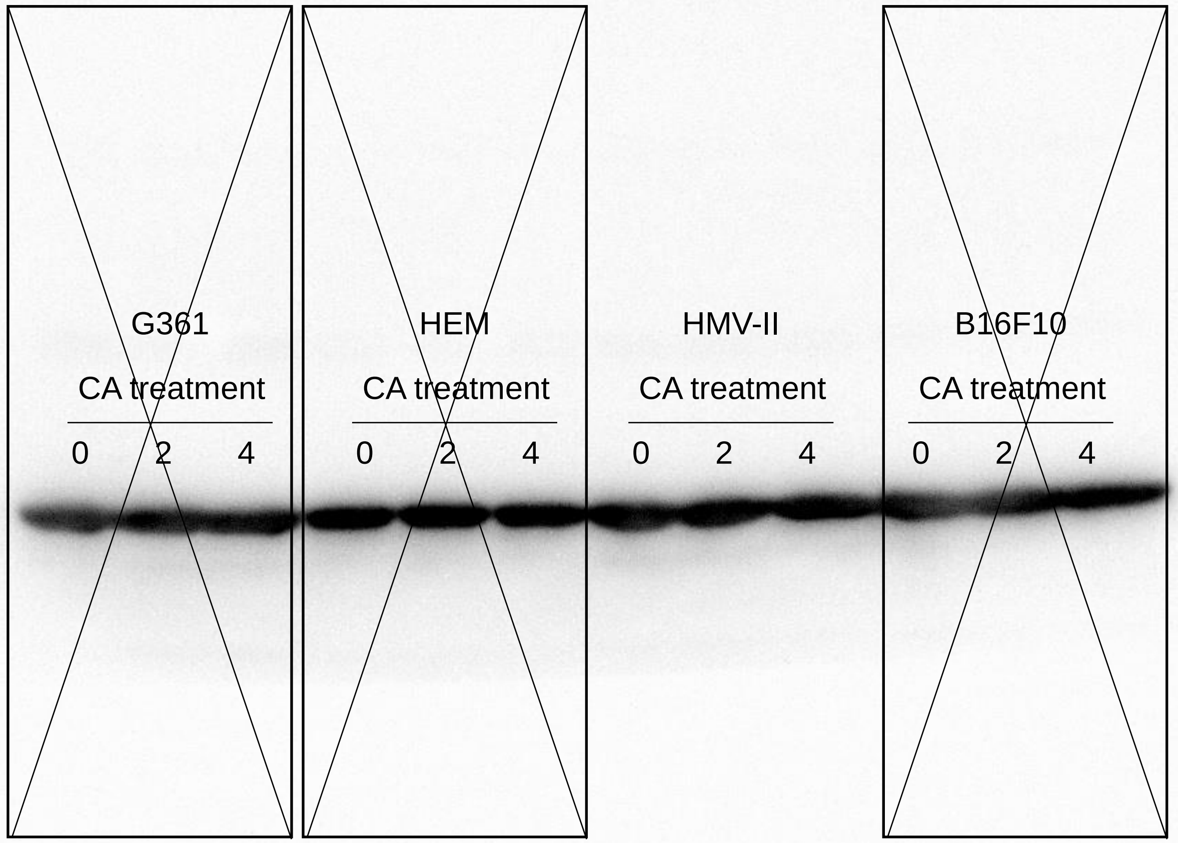

**Fig 4C MITF**

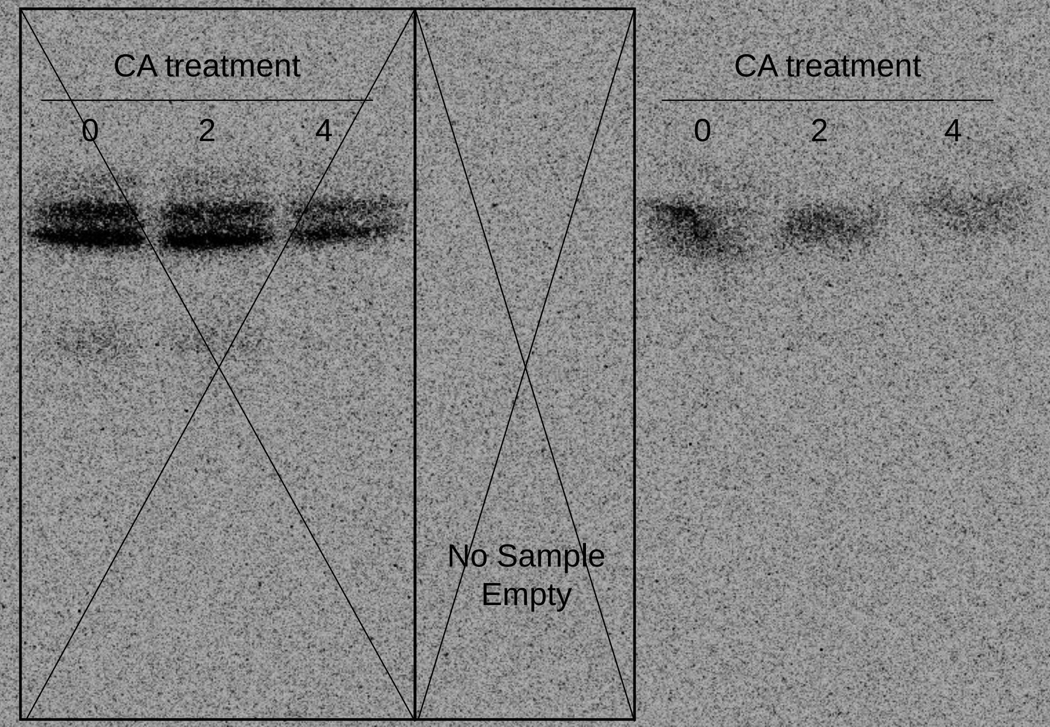

Fig 4C Tyrosinase (same proteins with MITF and β-actin, but on different gel)

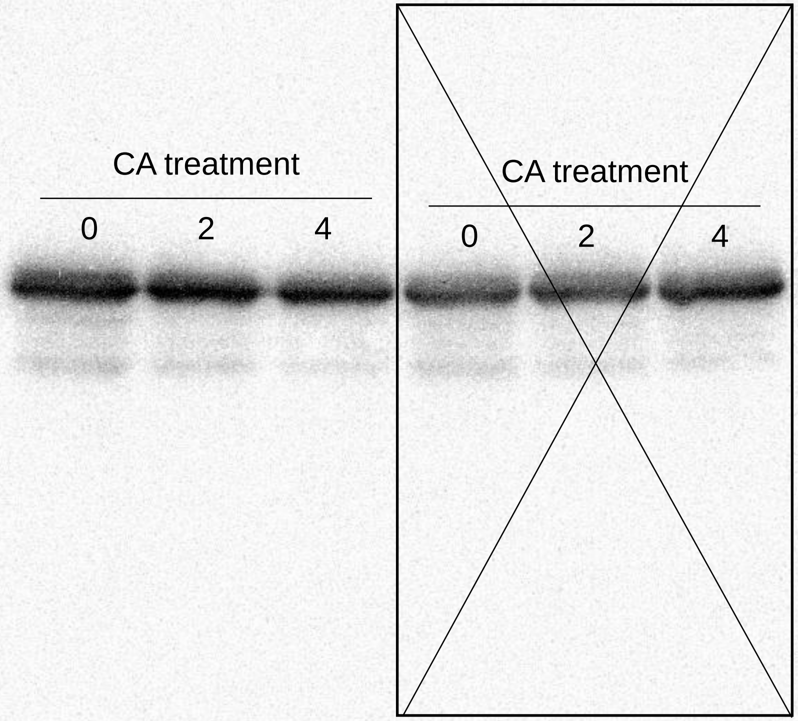

Fig 4C β-actin

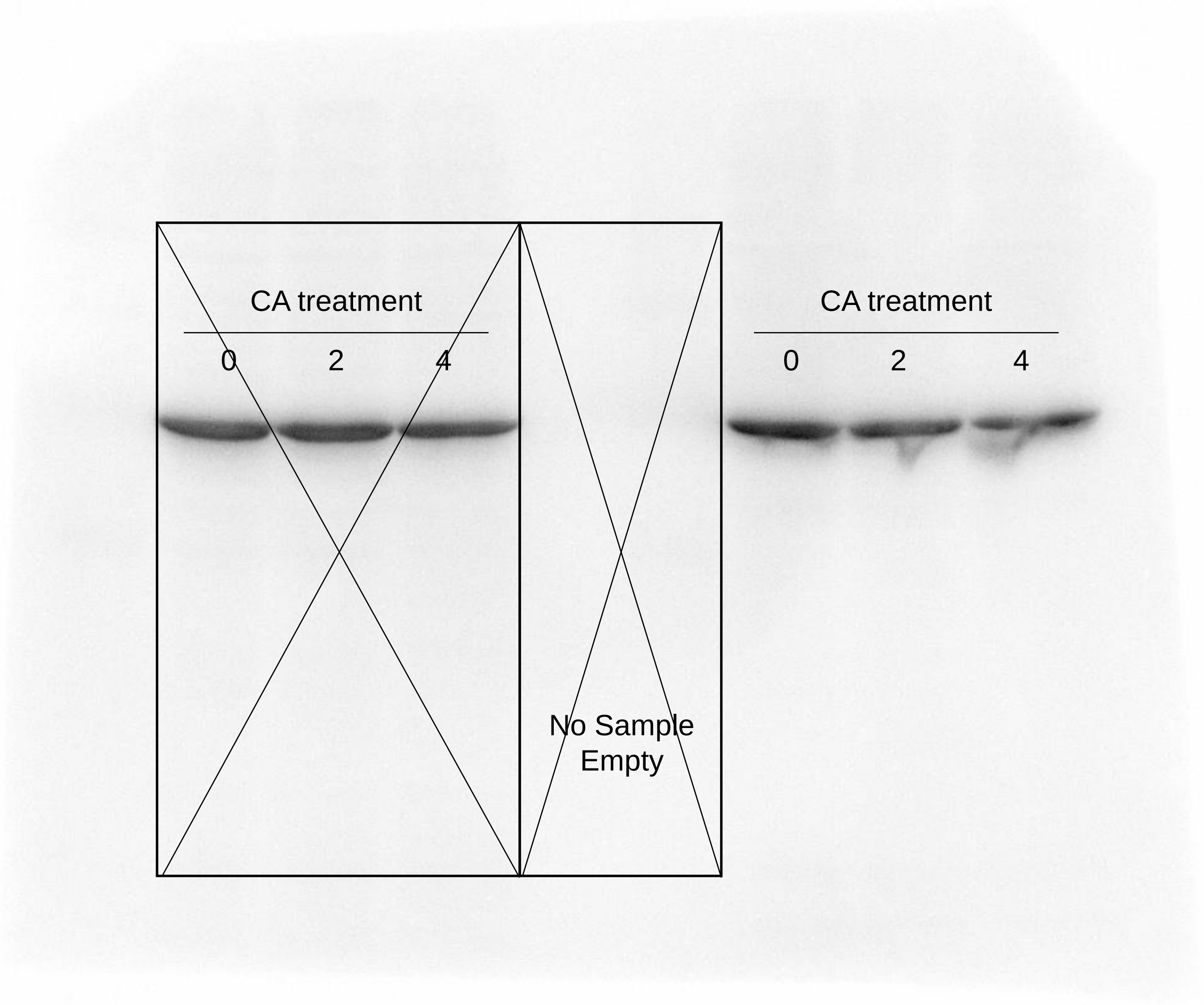

Fig 4C

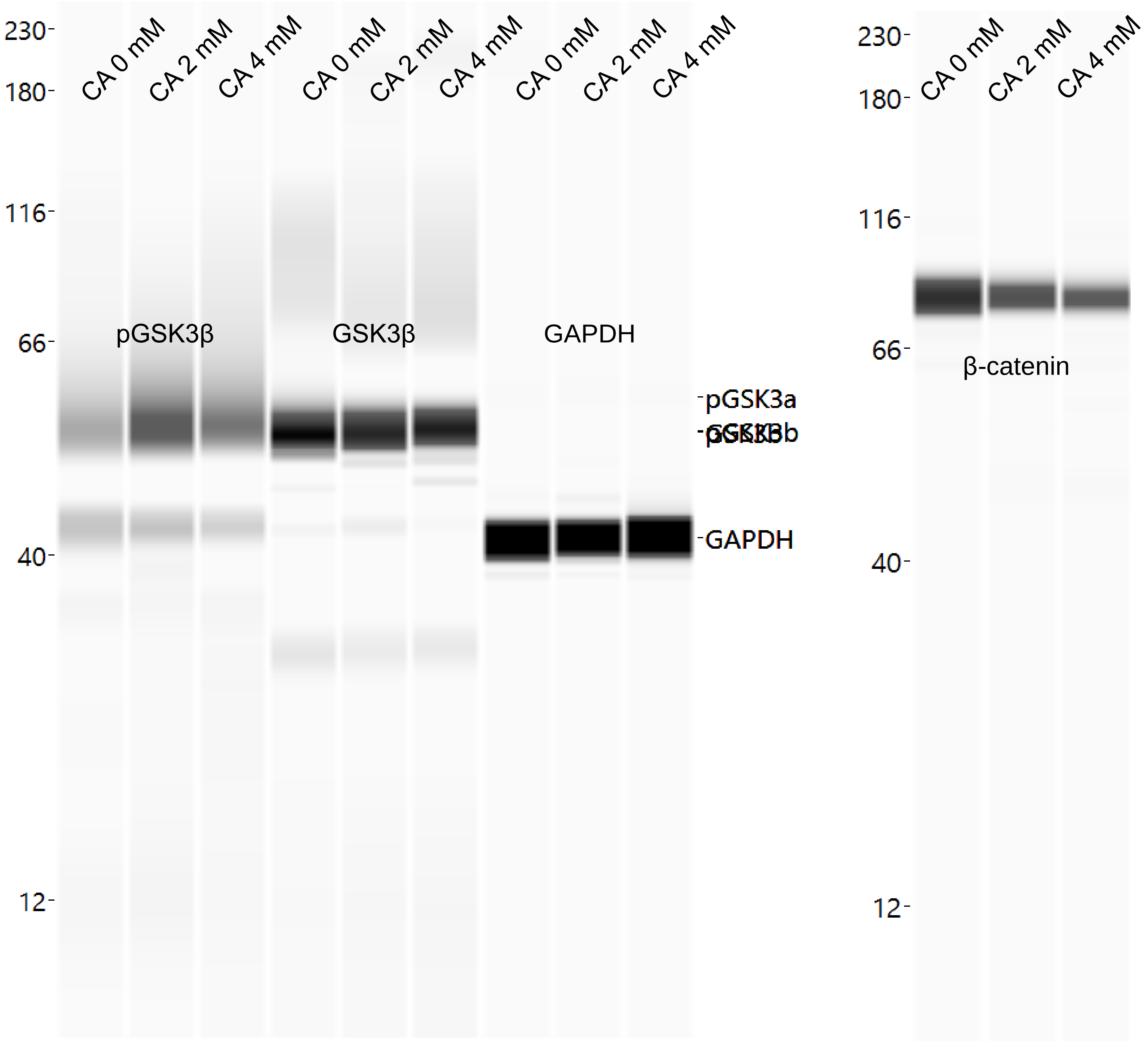

230
180
116
66
40
12
CA 0 mM
CA 2 mM
CA 4 mM
CA 0 mM
CA 2 mM
CA 4 mM
CA 0 mM
CA 2 mM
CA 4 mM
pGSK3β
GSK3β
GAPDH
pGSK3a
GAPDH
230
180
116
66
40
12
CA 0 mM
CA 2 mM
CA 4 mM
β-catenin

Fig 5B

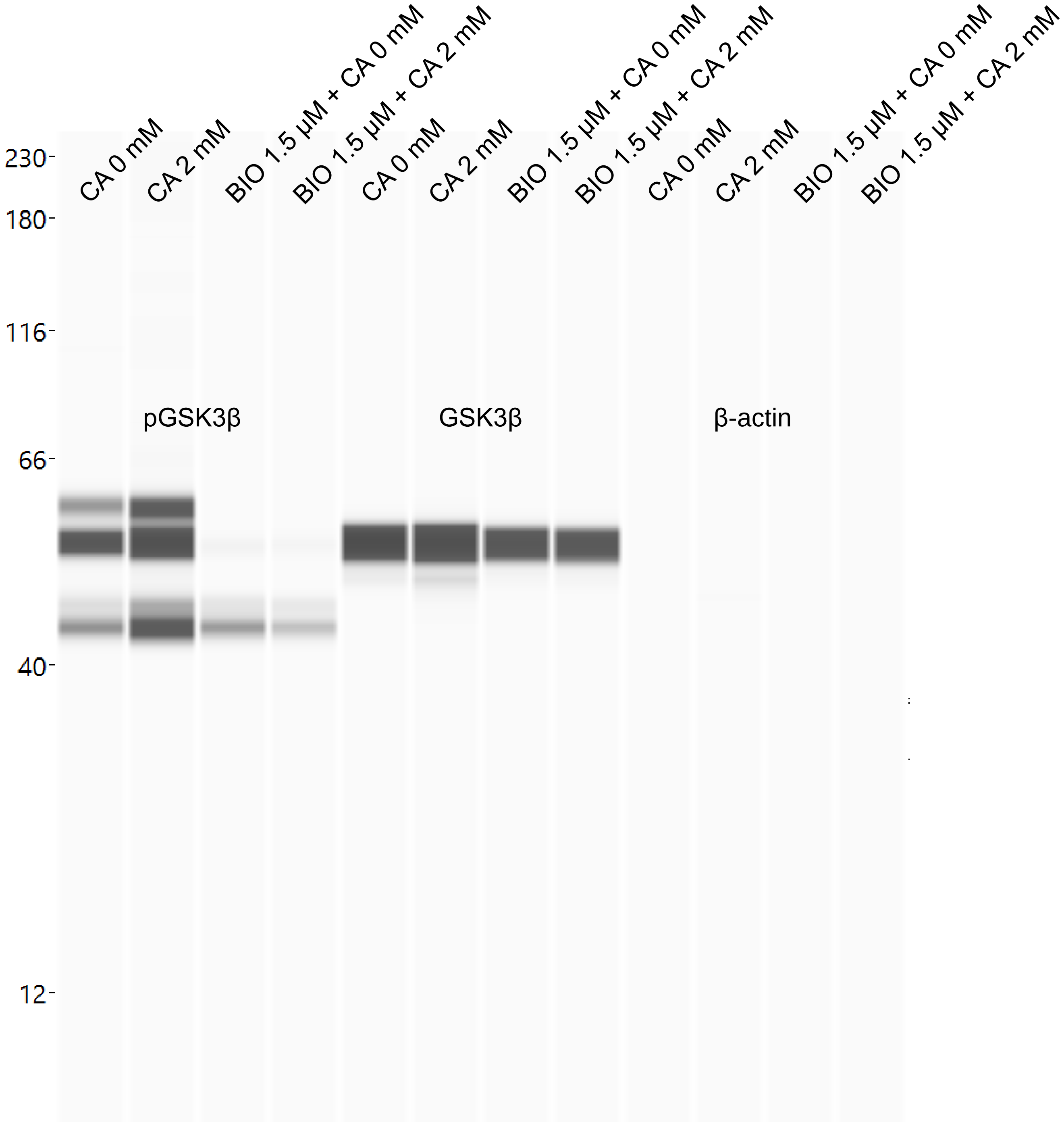

CA 0 mM
CA 2 mM
BIO 1.5 μM + CA 0 mM
BIO 1.5 μM + CA 2 mM
CA 0 mM
CA 2 mM
BIO 1.5 μM + CA 0 mM
BIO 1.5 μM + CA 2 mM
CA 0 mM
CA 2 mM
BIO 1.5 μM + CA 0 mM
BIO 1.5 μM + CA 2 mM
230
180
116
66
40
12
pGSK3β
GSK3β
β-actin

Fig 5B Contrast adjustment for β-actin

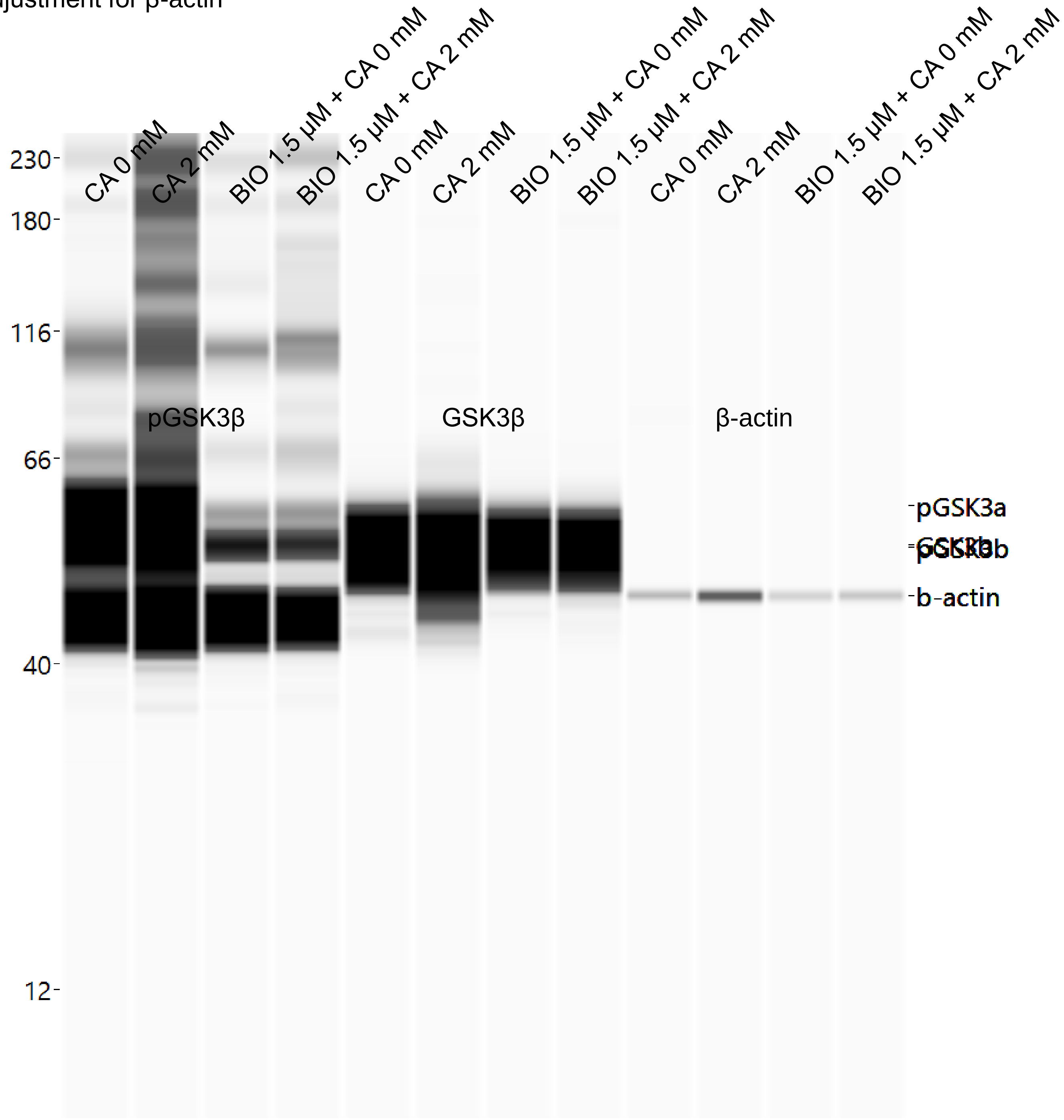

Fig 5B β-catenin

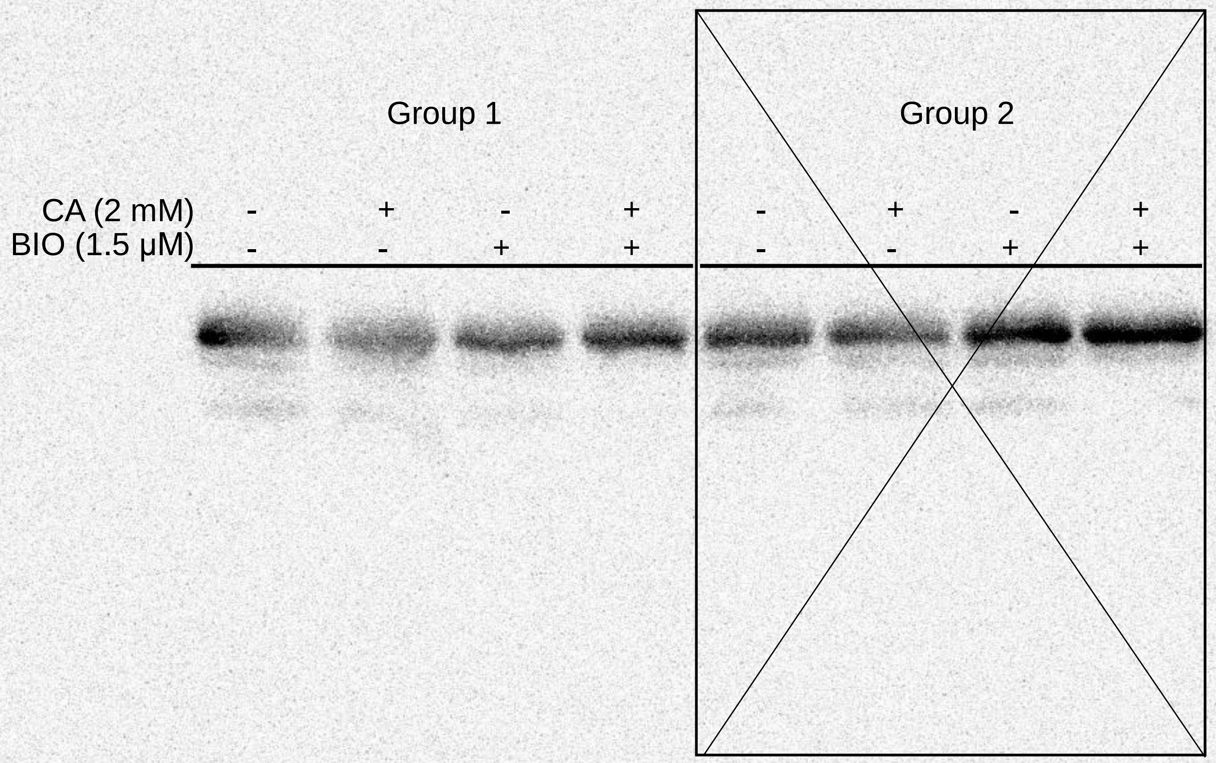

Fig 5B β-actin

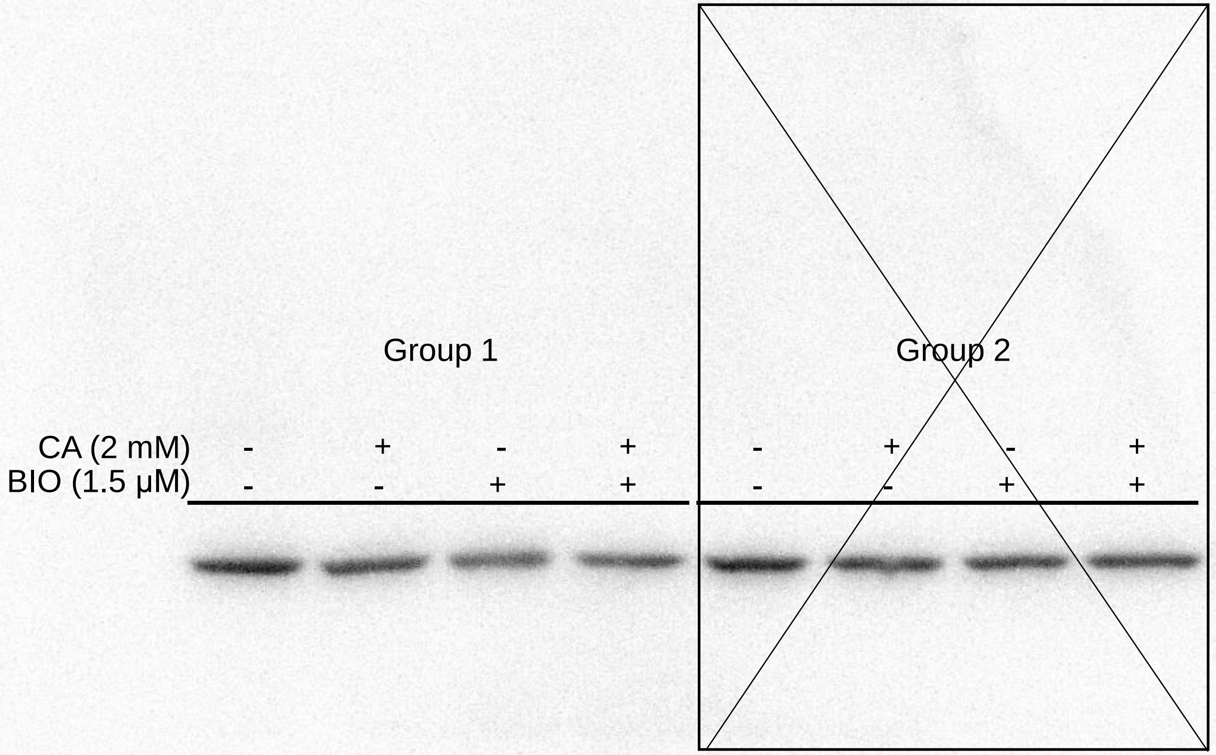

Fig 5D

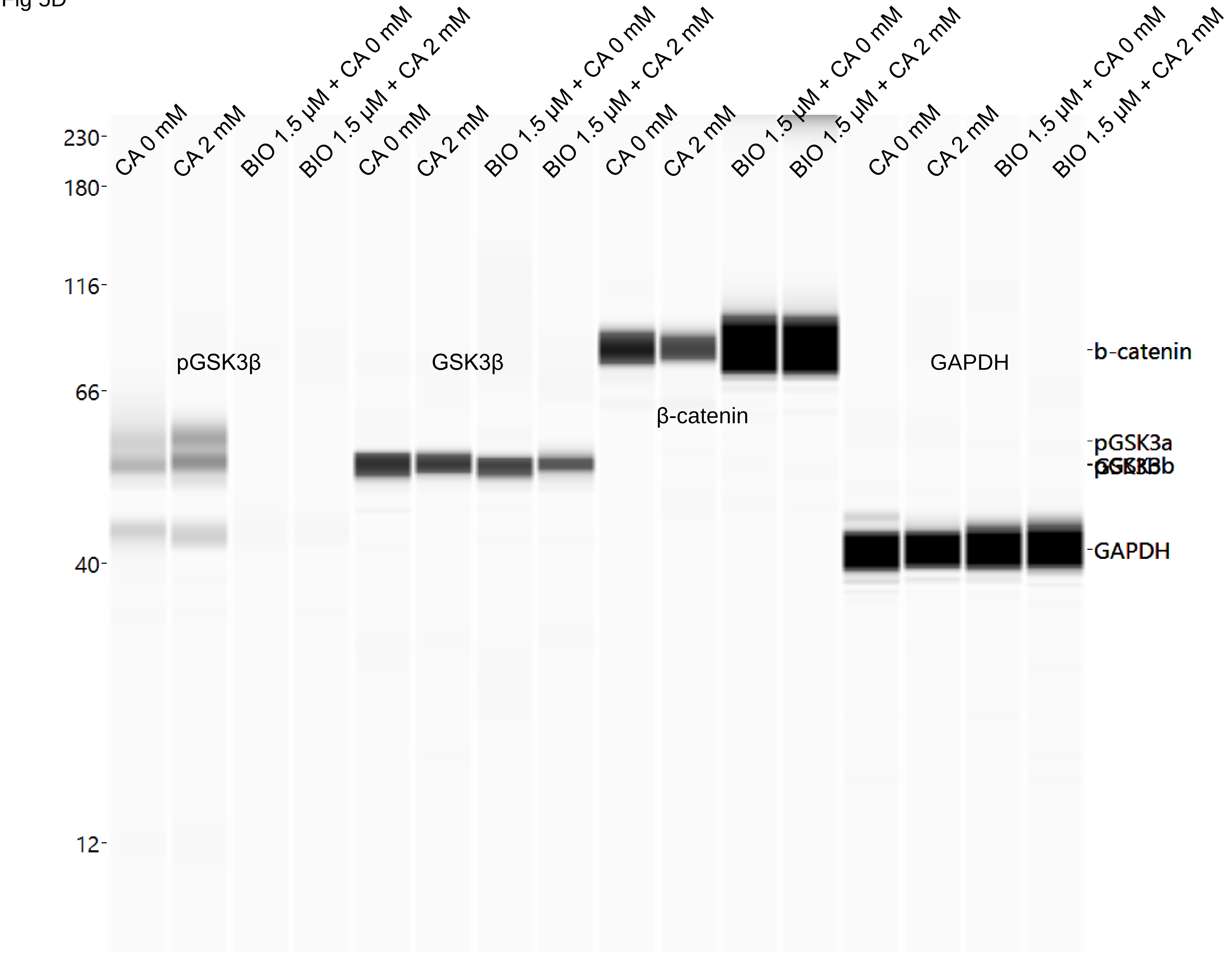

CA 0 mM
CA 2 mM
BIO 1.5 μM + CA 0 mM
BIO 1.5 μM + CA 2 mM
CA 0 mM
CA 2 mM
BIO 1.5 μM + CA 0 mM
BIO 1.5 μM + CA 2 mM
CA 0 mM
CA 2 mM
BIO 1.5 μM + CA 0 mM
BIO 1.5 μM + CA 2 mM
CA 0 mM
CA 2 mM
BIO 1.5 μM + CA 0 mM
BIO 1.5 μM + CA 2 mM
230
180
116
66
40
12
pGSK3β
GSK3β
β-catenin
GAPDH
b-catenin
pGSK3a
GAPDH
